# Three-Component Repurposed Technology for Enhanced Expression: Highly Accumulable Transcriptional Activators via Branched Tag Arrays

**DOI:** 10.1089/crispr.2018.0009

**Published:** 2018-10-23

**Authors:** Atsushi Kunii, Yoshihiro Hara, Mitsumasa Takenaga, Naoko Hattori, Takuya Fukazawa, Toshikazu Ushijima, Takashi Yamamoto, Tetsushi Sakuma

**Affiliations:** ^1^ Department of Mathematical and Life Sciences, Graduate School of Science, Hiroshima University, Hiroshima, Japan.; ^2^ Division of Epigenomics, National Cancer Center Research Institute, Tokyo, Japan.; ^3^ Department of General Surgery, Kawasaki Medical School, Okayama, Japan.

## Abstract

In the past few years, several types of artificial transcriptional activator, based on CRISPR-Cas9, have been developed and refined. Of these, in synergistic activation mediator and SunTag systems, the effector proteins, expressed in *trans*, can be recruited to the target sites via the MS2 RNA-binding system and GCN4-scFv antibody system, respectively. Here, we report a strong transcriptional activation system achieved by fusing GCN4 repeat to MS2 coat protein to accumulate numbers of activators, fused to scFv antibodies. By targeting the *CDH1* gene, we show that our novel system, named “TREE,” results in a greater effect of activating exogenous reporter and endogenous gene. Moreover, by targeting another gene, *RANKL*, we consistently show the superiority of the TREE system with fewer single-guide RNAs compared to conventional systems. Our TREE system is a promising tool for transcriptional activation and can potentially contribute to other dCas9-mediated technologies such as epigenome editing and chromosome visualization.

## Introduction

Recently, there has been rapid progress in research on the development and application of genome editing technology. As one of its derivative technologies, programmable regulation of gene expression has also been achieved in a site-specific manner. This system is particularly useful for direct cell reprogramming and for modeling and treating human diseases such as cancers.^1–5^ As the first-generation tool for activating specific gene expression (i.e., artificial transcriptional activators), an activation domain such as VP64 is fused to specific DNA-binding domains, including zinc-finger array, transcription activator-like effector, and catalytically inactive dCas9.^6–8^ Among them, the dCas9-based system is especially scalable because multiple sites can be simultaneously targeted by only expressing multiple single-guide RNAs (sgRNAs).

Based on this, several groups developed second-generation tools, which can mediate stronger activations. For example, the chimeric activator called “VPR” consisting of three types of activation mediator (VP64, p65, and Rta) was shown to have a stronger activation effect than VP64.^9^ Alternatively, activators could be provided in *trans* and recruited to the target sequences using RNA–protein or protein–protein interactions. In the synergistic activation mediator (SAM) system, modified sgRNAs harboring MS2 stem loops (sgRNA2.0) were used, and activators fused to MS2 coat proteins were recruited at the dCas9-VP64/sgRNA2.0-binding sites.^10^ In the “dCas9-SunTag” system, a repeat of GCN4 epitopes (SunTag) was connected to dCas9, which recruited the activators fused to scFv antibodies.^11^ It was also reported that extending the amino acid linkers connecting the individual GCN4 epitopes enabled a large epigenetic effector to induce DNA demethylation efficiently.^12^

Both the first- and the second-generation activator systems reportedly present a synergistic activation effect when multiple sgRNAs are used for single gene activation.^13,14^ In addition, in accordance with the previous comparative examination, although the SAM system often showed a superior effect among the second-generation systems, the system inducing the highest expression varied depending on the tested cell type, gene, and target sequence.^14^ These suggest that none of the second-generation systems has become a definitive one. In this study, to improve the efficiency of activation further by expanding the capacity for activator recruitment, a novel system named TREE (three-component repurposed technology for enhanced expression) was developed, and its functionality was compared to that of conventional systems.

## Materials and Methods

### Construction of sgRNA2.0 and dCas9-VP64 expression vectors

The previously established all-in-one CRISPR^*^-Cas9 vector system^15^ was modified to express sgRNA2.0 and dCas9-VP64. The sequence information is provided in the Supplementary Sequences (Supplementary Data are available online at www.liebertpub.com/crispr). To construct the gene-specific vectors, the oligonucleotides for the sgRNA templates, listed in the Supplementary Sequences, were annealed and inserted in accordance with a previously reported protocol.^16^ Subsequently, all required sgRNA and dCas9-VP64 expression cassettes were integrated into a single vector using Golden Gate assembly, following a previously described protocol.^17^

### Construction of MS2-effector and scFv-effector expression vectors

The cDNAs of MS2-p65-HSF1, VPR (VP64-p65-Rta), and scFv-sfGFP-VP64-GB1 were obtained from Addgene (plasmid #61423, #63798, and #60904). Then, cloning and substitutions were carried out by polymerase chain reaction (PCR) amplification and In-Fusion cloning (Takara, Shiga, Japan) as follows: MS2-p65-HSF1 cDNA was cloned into CMV-expression vector. For MS2-VPR, the sequence of p65-HSF1 was substituted by VPR. For scFv-p65-HSF1 and scFv-VPR, the cDNA of scFv-sfGFP- VP64-GB1 was cloned into CMV-expression vector. Then, the sequence of VP64 was substituted into p65-HSF1 or VPR. The coding sequences of MS2-effector and scFv-effector are described in the Supplementary Sequences.

### Construction of MS2-n× 22sTag and sgRNA/dCas9-n× 22sTag expression vectors

The cDNA of 4× 22sTag was synthesized by gBlocks (IDT, San Jose, CA). The synthesized sequence is described in the Supplementary Sequences. For MS2-4× and 8× 22sTag, one or two 4× 22sTag sequences were inserted downstream of MS2. For MS2-16 × and 24× 22sTag, the sequence of 8× 22sTag was amplified from MS2-8× 22sTag vector. Then, one or two of these sequences were inserted downstream of MS2-8× 22sTag. For dCas9-4× and 8× 22sTag, the sequences of 4× and 8× 22sTag were amplified from MS2-4× and 8× 22sTag vectors, respectively, and inserted downstream of dCas9. Insertions of sequences were performed using an In-Fusion HD Cloning Kit with the primers listed in the Supplementary Sequences. The coding sequences are also provided in the Supplementary Sequences.

### Construction of reporter vectors

The promoter and 5′ UTR regions of *CDH1* and *RANKL* were amplified from the genomic DNA collected from HEK293T cells. Then, these sequences were inserted upstream of *Luc2* cDNA (Promega, Madison, WI) using an In-Fusion HD Cloning Kit. The sequence information is provided in the Supplementary Sequences.

### Cell culture

MIA-PaCa2 cells were maintained in Dulbecco's modified Eagle's medium (DMEM; high glucose) with L-glutamine and Phenol Red (Wako, Osaka, Japan), supplemented with 10% fetal bovine serum (FBS; Thermo Fisher Scientific, Waltham, MA), 2.5% horse serum (Thermo Fisher Scientific), and 1% penicillin-streptomycin (Wako). HEK293T and HCT116 cells were maintained in DMEM (high glucose) supplemented with 10% FBS (Thermo Fisher Scientific), 1% minimum essential medium non-essential amino acids (Thermo Fisher Scientific), and 1% penicillin-streptomycin (Wako). All cell lines were tested negatively for mycoplasma contamination using an e-Myco Mycoplasma PCR Detection Kit (iNtRON Biotechnology, Seongnam, South Korea) and authenticated by short tandem repeat analysis (Takara).

### Transfection for the detection of MS2-22sTag proteins

A total of 60,000 cells were transfected with 200 ng of MS2-4 × , 8 × , 16 × , or 24 × 22sTag expression vector, or control pcDNA plasmid.

### Transfection for the reporter assays

A total of 60,000 cells were transfected with the vectors mixed as follows, using a Lipofectamine LTX reagent (Thermo Fisher Scientific) in a 96-well plate: a 1:1:1 mass ratio of the following three vectors: (1) sgRNAs/dCas9-effector all-in-one vector, sgRNAs/dCas9-*n*× 22sTag all-in-one vector, or pcDNA; (2) MS2-*n*× 22sTag expression vector, MS2-effector expression vector, or pcDNA; and (3) scFv-effector expression vector or pcDNA (50 ng in total for Figs. 2E and 3C and Supplementary Figs. S2C and S4B; 100 ng in total for Figs. 2C and D and 4A and C, and Supplementary Figs. S2C and S4B), and 100 and 20 ng of reporter vector and *R*Luc expression vector for reference, respectively.

### Transfection for the quantitative PCR and endogenous protein detection analyses

A total of 30,000 cells (for quantitative PCR) or 60,000 cells (for endogenous protein detection) were transfected with the vectors mixed as follows, using a Lipofectamine LTX reagent in a 96-well plate: a 1:1:1 mass ratio of the following three vectors: (1) sgRNAs/dCas9-effector all-in-one vector or pcDNA; (2) MS2-*n*× 22sTag expression vector, MS2-effector expression vector, or pcDNA; and (3) scFv-effector expression vector or pcDNA (200 ng in total). For the endogenous protein detection analyses, untransfected HCT116 cells were also used as positive controls, which were previously characterized as the *CDH1*-positive cells.^3^

### Transfection for the cytotoxicity analysis

A total of 30,000 cells were transfected with the vectors mixed as follows, using a Lipofectamine LTX reagent in a 96-well plate: a 1:1:1 mass ratio of the following three vectors: (1) non-targeting dCas9-effector expression vector or pcDNA; (2) MS2-*n*× 22sTag expression vector, MS2-effector expression vector, or pcDNA; and (3) scFv-effector expression vector or pcDNA (200 ng in total).

### Luciferase assay

At 24 h post transfection, dual luciferase activity was measured using a Dual-Glo Luciferase Assay System (Promega) on a TriStar LB 941 Multimode Microplate Reader (Berthold Technologies, Bad Wildbad, Germany).

### Analysis of endogenous *CDH1* and *RANKL* mRNA expression

At 48 h post transfection, cell lysis and reverse transcription were performed using a SuperPrep Cell Lysis & RT Kit for qPCR (Toyobo, Osaka, Japan), in accordance with the manufacturer's instructions. Relative mRNA expression levels were quantified by quantitative real-time PCR (qRT-PCR) using a KOD SYBR qPCR Mix (Toyobo) on a StepOnePlus Real-Time PCR System (Thermo Fisher Scientific). Expression levels of *CDH1* and *RANKL* were normalized by that of *RPL8*. Relative expression changes were calculated using the relative standard curve method. The primers used are listed in the Supplementary Sequences.

### Immunoblotting

At 24 h post transfection, the cells were collected and seeded onto a six-well plate. After 48 h, the cells were lysed and sonicated. Then, protein concentrations of lysates were measured using a Protein Assay Kit (Bio-Rad, Hercules, CA). The lysates were re-suspended in Laemmli buffer, denatured for 5 min at 98°C, and separated by Tris-glycine denaturing sodium dodecyl sulfate polyacrylamide gel electrophoresis. Proteins were blotted onto polyvinylidene fluoride membranes, blocked in 5% milk, and incubated overnight with the following primary antibodies: for endogenous protein detection, anti-E-cadherin (ab40772; Abcam, Cambridge, United Kingdom) or anti-α-tubulin (ab11304; Abcam); for MS2-22sTag protein detection, anti-HA (ab49969; Abcam) at a 1:1,000 (ab40772 and ab11304) or 1:2,000 (ab49969) dilution ratio in Can Get Signal Solution 1 (Toyobo) at 4°C. Subsequently, the proteins were incubated with the corresponding horseradish peroxidase–conjugated secondary antibodies (Thermo Fisher Scientific) at a 1:250 dilution ratio in Can Get Signal Solution 2 (Toyobo) for 1 h at room temperature. Chemiluminescent signals were generated using a SuperSignal West Pico Plus Chemiluminescent Substrate (Thermo Fisher Scientific) and captured on X-ray films (Fujifilm, Tokyo, Japan). The films were scanned, and signal intensities were quantified using ImageJ software.^*^

### Fluorescence immunocytochemistry

At 24 h post transfection, the cells were collected and seeded onto a 24-well plate. After 48 h, the cells were fixed with 4% paraformaldehyde in phosphate-buffered saline (PBS) for 15 min at room temperature. After washing with PBS, the cells were permeabilized with 1% Triton X-100 in PBS for 20 min at room temperature, and subsequently rinsed with PBS. The cells were covered with 1% bovine serum albumin in PBS for 1 h at room temperature, and were then incubated overnight with rabbit anti-E-cadherin (ab40772; Abcam) at a 1:100 dilution ratio at 4°C. After washing with PBS, the cells were stained with an Alexa 647-conjugated anti-rabbit secondary antibody (Thermo Fisher Scientific) at a 1:1,000 dilution ratio for 1 h at room temperature. After washing with PBS, nuclei were counterstained with DAPI. After washing with PBS, the images of DIC, DAPI, sfGFP, and E-cadherin (Alexa 647, pseudocolored red) were obtained with an FV-1000D confocal laser scanning microscope (Olympus, Tokyo, Japan).

### Cytotoxicity analysis

At 48 h post transfection, the cell viability was measured with ATP activity using a CellTiter-Glo Luminescent Cell Viability Assay (Promega) on a TriStar LB 941 Multimode Microplate Reader (Berthold Technologies) according to the manufacturers' instructions.

### Statistical analysis

All statistical analyses were performed with a Student's *t*-test.

## Results

### Concept of the TREE system

**FIG. 1.**
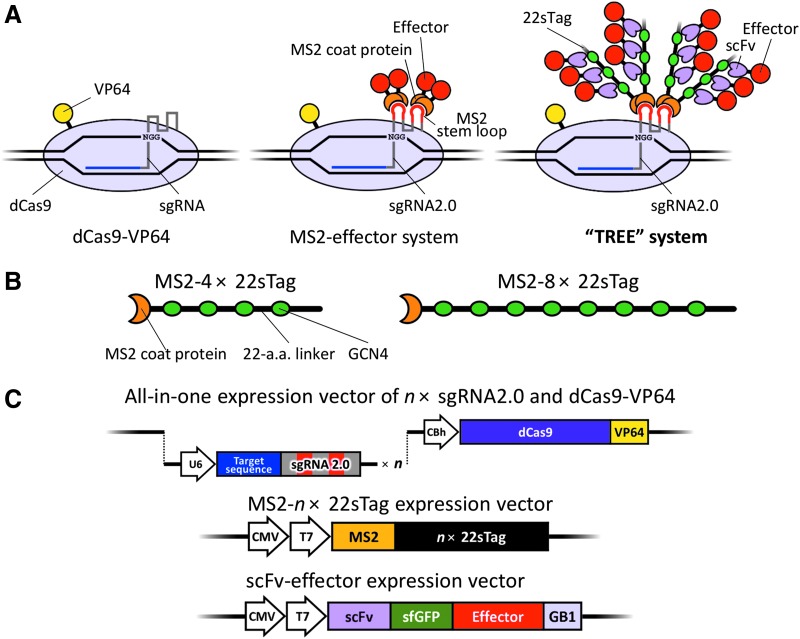
Schematic illustrations of the TREE (three-component repurposed technology for enhanced expression) systems. **(A)** Schematics of artificial transcriptional activator systems. Left: The first-generation system consisting of dCas9 fused with VP64 (dCas9-VP64) and sgRNA. Middle: The second-generation MS2-effector system. MS2 coat proteins directly fused to the effector molecules bind to MS2 stem loops of sgRNA 2.0. Right: The TREE system established in this study. MS2-22sTags are recruited as with the MS2-effector system. Then, scFv antibodies carrying effectors bind to GCN4 epitopes of 22sTags, theoretically resulting in the high accumulation of effector molecules around the target site. **(B)** Schematics of MS2-22sTag proteins. 4× or 8× repeats of GCN4 epitopes, each spaced with a 22 amino acid linker, are fused to MS2 coat proteins. The linkers at the bending positions illustrated consist of longer amino acids. **(C)** Schematics of the expression vectors to drive the TREE system. Top: Modified all-in-one CRISPR vector expressing multiple sgRNA2.0s and dCas9-VP64. Middle: MS2-*n*× 22sTag vectors. Bottom: scFv-effector vectors. p65-HSF1 or VPR was used as the effector.

In first-generation CRISPR-Cas9-based artificial transcriptional activators, an activation effector such as VP64 is directly fused to dCas9 (Fig. 1A, left).^8^ In contrast, in the most effective second-generation system, the SAM system, sgRNA2.0 is utilized to recruit transcriptional activators fused to MS2 coat proteins (Fig. 1A, middle).^10^ Here, we devised a tree-shaped, multiple-tag system to achieve stronger activation (Fig. 1A, right).

In our TREE system, sgRNA2.0 (root) and modified SunTag fused to MS2 coat protein (branch) were used as primary RNA tag and secondary peptide tag, respectively. On these branches, leaves (i.e., scFv effectors) were designed to bind, accumulating the transcriptional activation domains at the dCas9-binding sites. To construct the highly tandemized GCN4 repeat arrays, we initially synthesized 4× GCN4 repeat with 22 amino acid linkers, incorporating codon usage variations to avoid completely repeated DNA sequences. Then, each 4× repeat was assembled to create MS2-8× 22sTag (22 a.a.-spaced tag), as well as fusing MS2 coat protein (Fig. 1B and C, middle). Although we also tried to create MS2-16× and 24× 22sTag-expressing vectors and all four vectors were successfully constructed (Supplementary Fig. S1A), full-length proteins were not produced from the MS2-16× and 24× 22sTag vectors (Supplementary Fig. S1B). In the MS2-4× and 8× 22sTag vectors, full-length proteins were successfully expressed along with abundantly produced truncated proteins. Along with the construction of branch vectors, we created the root vector by modifying the previously established all-in-one CRISPR-Cas9 vector system.^15^ To optimize the vector for the TREE system, we repurposed the system to express multiple sgRNA2.0s and dCas9-VP64 simultaneously (Fig. 1C, top). As the leaf vector expressing *trans*-activator, we adopted an scFv-sfGFP-effector-GB1 framework, in accordance with a previous report (Fig. 1C, bottom).^11^ Regarding the effector, previously characterized chimeric activators, p65-HSF1 and VPR (VP64-p65-Rta), were used. p65-HSF1 was used in the SAM system with direct fusion to MS2 coat protein.^10^ VPR was previously used with direct fusion to dCas9 (dCas9-VPR).^9^ Using our 22sTag system, which has longer amino acid linkers than the original SunTag^11^ and has more epitope arrays than in the reports by Morita *et al*.,^12^ large effectors such as VPR are expected to accumulate at high levels and efficiently induce transcriptional activation.

### Characterization of the TREE system

**FIG. 2.**
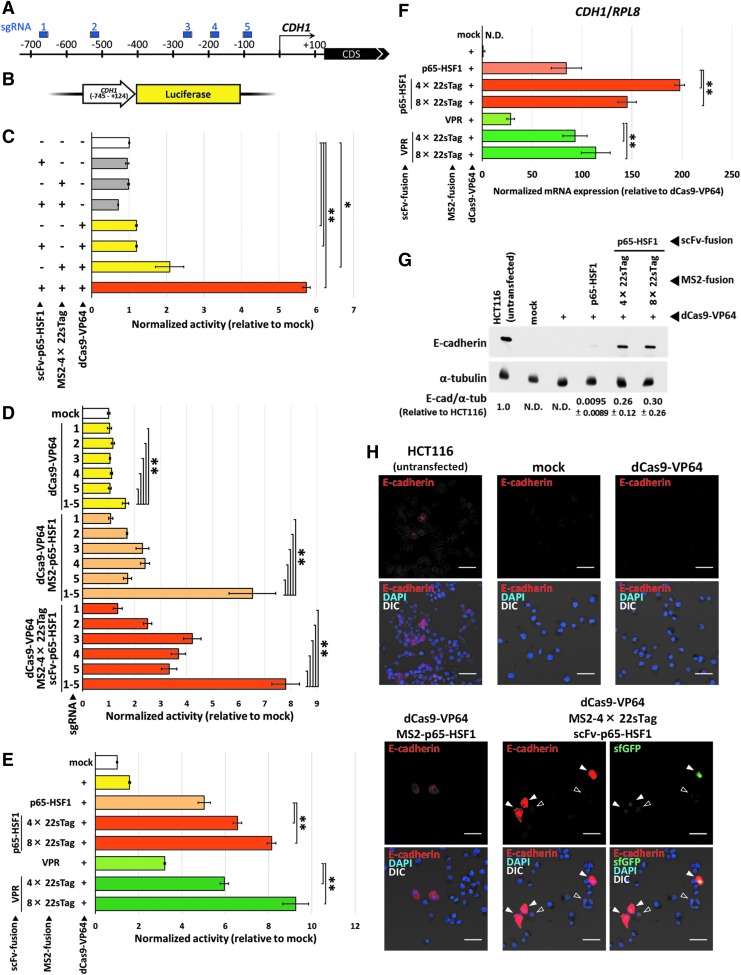
Activation of *CDH1* in MIA-PaCa2 cells with multiplex TREE system. **(A)** Schematic illustration of the positions of sgRNAs used for the activation of *CDH1*. Protospacer and protospacer adjacent motif (PAM) sequences are shown as blue and yellow boxes, respectively. See also Supplementary Figure S2A. **(B)** Schematic illustration of luciferase reporter vector containing *CDH1* promoter and 5stratio **(C)** Initial validation of the TREE system by reporter assay. All possible patterns of non-, single, double, and triple administration of the TREE components were tested. Data are shown as the mean ± standard deviation (*SD*; *n* = 4). **p < 0.01; *p < 0.05. **(D)** Activity comparison among single and five sgRNAs, using the dCas9-VP64, SAM, and TREE systems by the reporter assay. Data are shown as the mean ± *SD* (*n* = 4). **p < 0.01. **(E)** Activity comparison among the effectors and the repeat numbers of GCN4 epitopes, as well as the MS2-effector system, by reporter assay. Data are shown as the mean ± *SD* (*n* = 4). **p < 0.01; *p < 0.05. **(F)** Endogenous *CDH1* expression quantified by quantitative polymerase chain reaction (qPCR). Data are shown as the mean ± *SD* (*n* = 4). **p < 0.01; *p < 0.05. N.D., not detected. **(G)** Detection of E-cadherin and α-tubulin proteins by immunoblotting. Loaded protein mass is as follows: for the detection of E-cadherin in HCT116 and α-tubulin in all samples, 3 μg; for the detection of E-cadherin in all samples other than HCT116, 10 μg. Data are shown as the mean ± *SD* (*n* = 3), and one set of blots is shown. N.D., not detected. The other blots are shown in Supplementary Figure S3A. **(H)** Detection of E-cadherin protein by immunostaining. Filled and open triangles indicate E-cadherin/sfGFP- and sfGFP-positive cells, respectively. Scale bars, 50 μm. See also Supplementary Figure S3B.

To characterize the TREE system, we initially designed sgRNAs to activate the transcription of the human *CDH1* gene encoding the E-cadherin protein. The all-in-one vector expressing dCas9-VP64 and five sgRNAs targeting the promoter region of the *CDH1* gene (Fig. 2A and Supplementary Fig. S2A) was constructed, and a luciferase reporter vector containing the *CDH1* promoter and 5ed, and a luciferase reporter vector containing the ng the promoter region of the s targeti Fig. 2B). Regarding the cell line, we chose MIA-PaCa2 cells, in which the expression level of *CDH1* was reportedly quite low.^18^ We first checked the cytotoxicity of the TREE system with the comparison with the previous systems (Supplementary Fig. S2B). No significant cytotoxicity was observed in the TREE-introduced cells or in the dCas9-VP64-, SAM-, and dCas9-VPR-introduced cells. Subsequently, we tested basic mode of action of the TREE system by comparing the activation efficiency of the full set of TREE components to that of control groups lacking one or two components (Fig. 2C). As expected, weak and strong activation was observed in the dCas9-VP64/sgRNA2.0-expressing and all three vector-introduced groups, respectively.

Next, we investigated whether the conventional first- and second-generation systems and our TREE system showed stronger activation by simultaneously expressing five sgRNAs (Fig. 2D). In all three systems, dCas9-VP64, SAM (dCas9-VP64 and MS2-p65-HSF1), and our TREE system, the multiplex vectors showed statistically significant activation compared to the case with one sgRNA expression. In addition, even with only one sgRNA, the TREE system induced relatively high activities compared to the other systems. Subsequently, we checked the activity using the different numbers of GCN4 epitopes (4× and 8×) and different types of *trans*-activators (p65-HSF1 and VPR), as well as the conventional MS2-effector systems (Fig. 2E). All of the TREE vectors exhibited higher activity than the conventional second-generation MS2-effector vectors. Of these, especially high activation was observed in the samples in which the 8× 22sTag was used. Similar results were observed at different doses of plasmids (Supplementary Fig. S2C).

Following the results of the reporter assays, we attempted to activate endogenous *CDH1* expression in MIA-PaCa2 cells. qRT-PCR analysis revealed that the first-generation dCas9-VP64 could hardly activate transcription, while the conventional second-generation MS2-effector systems and our TREE systems could upregulate the transcriptional level (Fig. 2F). Notably, all TREE vectors showed a significantly stronger effect than the MS2-effector vectors, although the relationship of the activity levels among the variations of the TREE systems differed from that observed in the reporter assay (e.g., the activation levels using scFv-p65-HSF1 and scFv-VPR were comparable in the reporter assay, while scFv-p65-HSF1 was better than scFv-VPR in the qRT-PCR analysis). We subsequently quantified the protein level of E-cadherin by immunoblotting (Fig. 2G and Supplementary Fig. S3A). The signals of E-cadherin protein were invisible and slightly visible in the dCas9-VP64- and SAM-introduced samples, respectively, while they were intense in the TREE-introduced samples. Induced expression of E-cadherin protein was also confirmed by immunostaining (Fig. 2H and Supplementary Fig. S3B). Consistent with the results of immunoblotting analysis, the fluorescence signals were almost invisible in mock- and dCas9-VP64-transfected cells, whereas the E-cadherin-positive cells emerged in the groups transfected with the MS2-effector and TREE systems, with a tendency for stronger fluorescence in the TREE-introduced cells than in the SAM-introduced ones. Notably, in the TREE systems, we could directly observe the TREE component-expressing cells by monitoring the green fluorescence derived from sfGFP. Although some of the E-cadherin-positive cells did not show visible green fluorescence (Supplementary Fig. S3B), possibly because of the low intensity of fluorescence of sfGFP fused with various domains, most of the clearly visible sfGFP-positive cells showed highly upregulated E-cadherin signals (Fig. 2H), suggesting that the upregulation of E-cadherin correctly occurred in the transfected cells.

### Target gene- and cell type-independent superiority of the TREE system

**FIG. 3.**
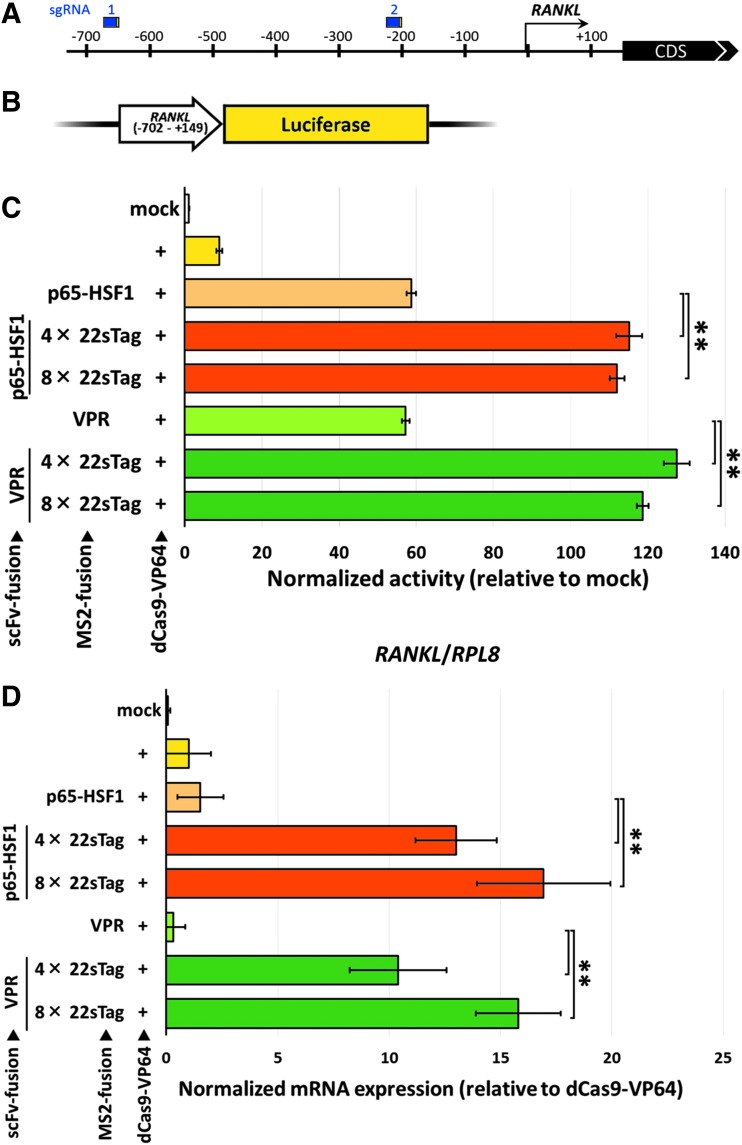
Activation of *RANKL* in HEK293T cells with multiplex TREE system. **(A)** Schematic illustration of the positions of sgRNAs used for the activation of *RANKL*. Protospacer and PAM sequences are shown as blue and yellow boxes, respectively. See also Supplementary Figure S4A. **(B)** Schematic illustration of luciferase reporter vector containing *RANKL* promoter and 5stratio **(C)** Activity comparisons among the effectors and the repeat numbers of GCN4 epitopes, as well as the MS2-effector system, by the reporter assay. Data are shown as the mean ± *SD* (*n* = 4). **p < 0.01. **(D)** Endogenous *RANKL* expression quantified by qPCR. Data are shown as the mean ± *SD* (*n* = 4). **p < 0.01.

To investigate whether the superiority of our TREE system is target gene- or cell line-specific, we targeted another gene, *RANKL* (*TNFSF11*), in another cell line, HEK293T. Previously, the transcriptional activation of *RANKL* using dCas9-TET1 and MS2-TET1 was reported.^19^ We chose two of the sgRNAs shown as the most effective in the corresponding paper, targeting 700 and 200 bp upstream of the transcription start site (TSS; Fig. 3A and Supplementary Fig. S4A), although these designs might be suboptimal for activator-mediated enhancement of expression because the activity range of SAM activators, for example, was shown to be maximally active in the −100 to 0 TSS range.^10^ Then, we constructed a reporter vector containing both target sequences (Fig. 3B) and an all-in-one CRISPR vector expressing two sgRNAs and dCas9-VP64, similar to those for the *CDH1* locus.

Consistent with the results of *CDH1* activation, the *RANKL* reporter assay revealed that our TREE systems showed higher activity than the conventional second-generation MS2-effector systems (Fig. 3C), and the dose-dependent effects were also confirmed (Supplementary Fig. S4B). Critically, endogenous expression of *RANKL* mRNA was not significantly activated using both conventional first- and second-generation systems, while our TREE systems could achieve highly upregulated transcription of the endogenous *RANKL* gene (Fig. 3D). Note that the *RANKL* sgRNAs were suboptimally designed as described above. However, our TREE systems could act as the strong transcriptional activators, even using such sgRNAs. On the other hand, we found that our TREE system slightly decreased cell viability in HEK293T cells, inconsistent with the results obtained using MIA-PaCa2 cells (Supplementary Fig. S4C).

Additionally, we performed a direct comparison of our TREE systems and another second-generation system, dCas9-22sTag fusion, similar to the SunTag, in the *RANKL* reporter assay. We additionally constructed two types of all-in-one CRISPR vector, expressing two sgRNAs without MS2 stem loops targeting the *RANKL* promoter and dCas9-4× 22sTag or -8× 22sTag, instead of dCas9-VP64. Thorough analysis of the expression-enhancing activity revealed the clear superiority of our TREE systems over the dCas9-22sTag systems (Supplementary Fig. S4D).

### Superiority of the TREE system with only one sgRNA

**FIG. 4.**
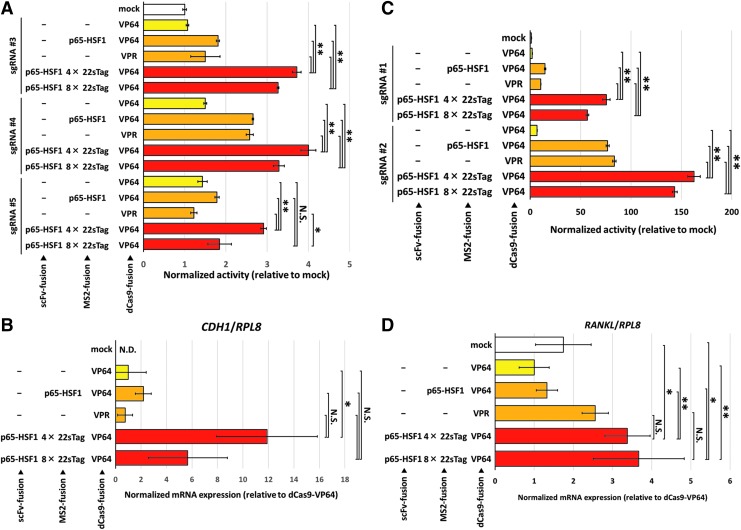
Robust transcriptional activation with single TREE system. **(A)** Assessment of the activation capacity of single TREE systems containing sgRNA #3, #4, or #5 by *CDH1* reporter assay in MIA-PaCa2 cells with the comparison with the previous systems. Data are shown as the mean ± *SD* (*n* = 4). **p < 0.01; *p < 0.05. N.S., not significant. **(B)** Assessment of the activation capacity of single TREE systems containing sgRNA #5 by qPCR analysis of endogenous *CDH1* gene in MIA-PaCa2 cells with the comparison with the previous systems. Data are shown as the mean ± *SD* (*n* = 3). *p < 0.05. N.S., not significant; N.D., not detected. **(C)** Assessment of the activation capacity of single TREE systems containing sgRNA #1 or #2 by *RANKL* reporter assay in HEK293T cells with the comparison with the previous systems. Data are shown as the mean ± *SD* (*n* = 4). **p < 0.01. **(D)** Assessment of the activation capacity of single TREE systems containing sgRNA #2 by qPCR analysis of endogenous *RANKL* gene in HEK293T cells with the comparison with the previous systems. Data are shown as the mean ± *SD* (*n* = 4). **p < 0.01; *p < 0.05. N.S., not significant. For dCas9-VPR, sgRNAs without MS2 stem loops were used instead of sgRNA2.0.

Finally, we investigated the superiority of our TREE system to the conventional dCas9-VP64, SAM, and dCas9-VPR technologies with only one sgRNA. We first selected three sgRNAs, sgRNA #3–5, for the activation of *CDH1*, based on the activation scores determined in Figure 2D. Reporter assays revealed that all the TREE vectors with one sgRNA outperformed SAM and dCas9-VPR systems, with the exception that the activation score of MS2-8 × 22sTag-containing TREE system with sgRNA #5 was comparable to that of dCas9-VP64 and SAM system (Fig. 4A). One sgRNA-derived transcriptional activation was further characterized by endogenous qPCR analysis. Using sgRNA #5, the overall relationship of activation levels with each system was quite similar to that observed in the reporter assays (Fig. 4B). Importantly, the average score of MS2-8 × 22sTag-containing TREE system was about sixfold greater than that of SAM system, although statistical significance could not be confirmed because of the score variability.

Similar to the *CDH1* locus, we constructed the sgRNA #1 or #2-expressing dCas9-VP64, SAM, and dCas9-VPR, and validated their functionality by reporter assay and qPCR analysis. Significant reporter activation was observed in all the TREE vectors constructed compared to the conventional systems (Fig. 4C). It is particularly noteworthy that our TREE vector containing sgRNA #1 retained about a half activity of sgRNA #2-containing TREE vector, while none of the previous systems resulted in high-level activation. Our TREE vectors also highly activated the endogenous *RANKL* with sgRNA #2 (Fig. 4D), and their average activation levels were higher than those with previous systems. Statistically significant difference of TREE versus previous systems was also observed, except TREE versus dCas9-VPR.

## Discussion

In summary, we established a novel hybrid system of the previously characterized SAM and SunTag activators, enabling sequential recruitment of the tag arrays and effector molecules, and showed high-powered transcriptional activation efficacy at two gene loci in different cell lines. Our achievements are somewhat contrary to a previous paper by Chavez *et al*. reporting that various combinations of second-generation systems (e.g., dCas9-10 × GCN4 + sgRNA2.0 + MS2-p65-HSF1 + scFv-VP64) did not show any additive or synergistic activation effects.^14^ One possible explanation for this contradiction is that a simple “addition” of various systems was not effective for further accumulation of the effector molecules, but our TREE-shaped hierarchical configuration (i.e., “integration” of SAM and SunTag systems) was effective to highly accumulate trans-activators. Chavez *et al*. just collectively used the previous systems, while we built the high-order system by using newly created MS2-22sTag proteins as the adapter molecules. In addition, there is still room for improvement in optimizing the expression levels of the TREE components, which might result in greater efficiency of transcriptional activation. In fact, we set the mass ratio of three TREE vectors as 1:1:1 throughout this study, but this ratio might be suboptimal because every “root” (MS2 stem loop) requires two “branches” (MS2-22sTag proteins) and every “branch” requires either four or eight “leaves” (scFv-activators). Thus, further optimization in terms of the stoichiometry of these components will be required to achieve maximum level of activation.

Moreover, based on their architecture, our TREE system might overcome the obstacles that were difficult to solve using the existing methods by exploiting its particular attributes. For example, the conventional MS2-effector system has the ability to distribute several types of effector to independent target loci by using multiple types of RNA–protein interaction.^20,21^ However, this system has a limit on the number of recruitable effector molecules. In contrast, the dCas9-SunTag system can accumulate more effectors, but it cannot discriminate each locus to assign various effectors. In contrast, using our TREE system, highly accumulated recruitment of different effectors would be achieved in a locus-specific manner, by simultaneously using the 22sTag and another tag proteins fused with multiple RNA-binding proteins (e.g., MS2-22sTag and PP7-another tag).

Another anticipated application of the TREE system is targeted gene activation *in vivo*. Recently, gene activation in mice with adeno-associated virus (AAV)-mediated delivery of a SAM-like system was reported.^22^ AAV has a strict size limit. Thus, direct fusion of large tag arrays or effectors such as SunTag or VPR is not applicable in AAV-mediated delivery. However, such tags and effectors can be supplied independently in our TREE system, in which the lengths of MS2-22sTag and scFv-effector cDNAs are capable of loading in AAV: MS2-4 × 22sTag, 1,218 bp; MS2-8 × 22sTag, 1,710 bp; scFv-p65-HSF1, 2,823 bp; scFv-VPR, 3,441 bp (Supplementary Sequences). Therefore, our system would also be suitable for *in vivo* application, although the actual applicability has not yet experimentally confirmed.

Our investigations also revealed some challenges and open questions. First, the DNA and amino acid sequences of MS2-22sTag might be reconsidered to achieve further enhancement in accordance with the Western blotting analysis of the corresponding proteins. In previous papers reporting original and modified SunTag systems,^11,12^ their protein expression was not examined. Therefore, it should be clarified whether this phenomenon is 22sTag-specific. Second, the cytotoxicity analyses of the TREE system using two cell lines resulted in different outcomes. No significant toxicity was observed in MIA-PaCa2 cells, whereas the system components showed slight toxicity in HEK293T cells. This cell type–specific toxicity should be further characterized. Third, both the sufficient level of transcriptional activation to induce protein upregulation and a ceiling to the level of transcriptional activation regardless of the basal expression of the target transcript, observed in the previous paper,^10^ were not examined in the context of this study. Fourth, the comparative analyses between our TREE system and the previous systems should be more thoroughly examined at various target loci in various cell lines to confirm the robust superiority of our system further. Especially with only one sgRNA, there was high variability in endogenous gene expression with the TREE activation. Such variability should be caused by the lack of tight robustness of the current TREE system with one sgRNA. Therefore, the robust activation not with the multiple TREE but with the single TREE will be the future avenue of this technology. Fifth, the specificity of this system should be assessed by comprehensive RNA-seq analysis. These points should be clarified in the future study.

Potential application of the TREE system is not limited to simple transcriptional activation. The MS2- or SunTag-mediated accumulation of various molecules has already been reported in various applications, including targeted DNA demethylation,^12,19^ targeted histone modification,^23^ visualization of specific chromosomal regions,^24^ and directed evolution with saturation mutagenesis.^25^

## Conclusion

Our TREE system not only has the potential to be a promising system of artificial transcriptional activator but also would contribute to a broad range of biological analyses assisted by the CRISPR system with various effectors, boosting and adding depth to life science studies.

## Supplementary Material

Supplemental data

Supplemental data

Supplemental data

Supplemental data

Supplemental data

## Notes

^*^ ***C****lustered* ***R****egularly* ***I****nterspaced* ***S****hort* ***P****alindromic* ***R****epeats.*

^*^ https://imagej.nih.gov/ij/
