# Supplementary material for: Three-Component Repurposed Technology for Enhanced Expression: Highly Accumulable Transcriptional Activators via Branched Tag Arrays
Source: CRISPR J. 2018 Oct 23;1(5):337–47. doi: 10.1089/crispr.2018.0009 (PMC6636879; doi:10.1089/crispr.2018.0009)
Supplement: Supplemental data [file Supp_Data.docx]

**Supplementary Sequences**

**sgRNA 2.0**

**NNNNNNNNNNNNNNNNNNNN**GTTTTAGAGCTAGGCCA**ACATGAGGATCACCCATGT**CTGCAGGGCCTAGCAAGTTAAAATAAGGCTAGTCCGTTATCAACTTGGCCA**ACATGAGGATCACCCATGT**CTGCAGGGCCAAGTGGCACCGAGTCGGTGCTTTTTTT

**3xFLAG-SV40 NLS-dCas9-Nuleoplasmin NLS-VP64**

**GACTATAAGGACCACGACGGAGACTACAAGGATCATGATATTGATTACAAAGACGATGA**CGATAAGATGGCC**CCAAAGAAGAAGCGGAAGGTC**GGTATCCACGGAGTCCCAGCAGCC**GACAAGAAGTACAGCATCGGCCTGGCCATCGGCACCAACTCTGTGGGCTGGGCCGTGATCACCGACGAGTACAAGGTGCCCAGCAAGAAATTCAAGGTGCTGGGCAACACCGACCGGCACAGCATCAAGAAGAACCTGATCGGAGCCCTGCTGTTCGACAGCGGCGAAACAGCCGAGGCCACCCGGCTGAAGAGAACCGCCAGAAGAAGATACACCAGACGGAAGAACCGGATCTGCTATCTGCAAGAGATCTTCAGCAACGAGATGGCCAAGGTGGACGACAGCTTCTTCCACAGACTGGAAGAGTCCTTCCTGGTGGAAGAGGATAAGAAGCACGAGCGGCACCCCATCTTCGGCAACATCGTGGACGAGGTGGCCTACCACGAGAAGTACCCCACCATCTACCACCTGAGAAAGAAACTGGTGGACAGCACCGACAAGGCCGACCTGCGGCTGATCTATCTGGCCCTGGCCCACATGATCAAGTTCCGGGGCCACTTCCTGATCGAGGGCGACCTGAACCCCGACAACAGCGACGTGGACAAGCTGTTCATCCAGCTGGTGCAGACCTACAACCAGCTGTTCGAGGAAAACCCCATCAACGCCAGCGGCGTGGACGCCAAGGCCATCCTGTCTGCCAGACTGAGCAAGAGCAGACGGCTGGAAAATCTGATCGCCCAGCTGCCCGGCGAGAAGAAGAATGGCCTGTTCGGAAACCTGATTGCCCTGAGCCTGGGCCTGACCCCCAACTTCAAGAGCAACTTCGACCTGGCCGAGGATGCCAAACTGCAGCTGAGCAAGGACACCTACGACGACGACCTGGACAACCTGCTGGCCCAGATCGGCGACCAGTACGCCGACCTGTTTCTGGCCGCCAAGAACCTGTCCGACGCCATCCTGCTGAGCGACATCCTGAGAGTGAACACCGAGATCACCAAGGCCCCCCTGAGCGCCTCTATGATCAAGAGATACGACGAGCACCACCAGGACCTGACCCTGCTGAAAGCTCTCGTGCGGCAGCAGCTGCCTGAGAAGTACAAAGAGATTTTCTTCGACCAGAGCAAGAACGGCTACGCCGGCTACATTGACGGCGGAGCCAGCCAGGAAGAGTTCTACAAGTTCATCAAGCCCATCCTGGAAAAGATGGACGGCACCGAGGAACTGCTCGTGAAGCTGAACAGAGAGGACCTGCTGCGGAAGCAGCGGACCTTCGACAACGGCAGCATCCCCCACCAGATCCACCTGGGAGAGCTGCACGCCATTCTGCGGCGGCAGGAAGATTTTTACCCATTCCTGAAGGACAACCGGGAAAAGATCGAGAAGATCCTGACCTTCCGCATCCCCTACTACGTGGGCCCTCTGGCCAGGGGAAACAGCAGATTCGCCTGGATGACCAGAAAGAGCGAGGAAACCATCACCCCCTGGAACTTCGAGGAAGTGGTGGACAAGGGCGCTTCCGCCCAGAGCTTCATCGAGCGGATGACCAACTTCGATAAGAACCTGCCCAACGAGAAGGTGCTGCCCAAGCACAGCCTGCTGTACGAGTACTTCACCGTGTATAACGAGCTGACCAAAGTGAAATACGTGACCGAGGGAATGAGAAAGCCCGCCTTCCTGAGCGGCGAGCAGAAAAAGGCCATCGTGGACCTGCTGTTCAAGACCAACCGGAAAGTGACCGTGAAGCAGCTGAAAGAGGACTACTTCAAGAAAATCGAGTGCTTCGACTCCGTGGAAATCTCCGGCGTGGAAGATCGGTTCAACGCCTCCCTGGGCACATACCACGATCTGCTGAAAATTATCAAGGACAAGGACTTCCTGGACAATGAGGAAAACGAGGACATTCTGGAAGATATCGTGCTGACCCTGACACTGTTTGAGGACAGAGAGATGATCGAGGAACGGCTGAAAACCTATGCCCACCTGTTCGACGACAAAGTGATGAAGCAGCTGAAGCGGCGGAGATACACCGGCTGGGGCAGGCTGAGCCGGAAGCTGATCAACGGCATCCGGGACAAGCAGTCCGGCAAGACAATCCTGGATTTCCTGAAGTCCGACGGCTTCGCCAACAGAAACTTCATGCAGCTGATCCACGACGACAGCCTGACCTTTAAAGAGGACATCCAGAAAGCCCAGGTGTCCGGCCAGGGCGATAGCCTGCACGAGCACATTGCCAATCTGGCCGGCAGCCCCGCCATTAAGAAGGGCATCCTGCAGACAGTGAAGGTGGTGGACGAGCTCGTGAAAGTGATGGGCCGGCACAAGCCCGAGAACATCGTGATCGAAATGGCCAGAGAGAACCAGACCACCCAGAAGGGACAGAAGAACAGCCGCGAGAGAATGAAGCGGATCGAAGAGGGCATCAAAGAGCTGGGCAGCCAGATCCTGAAAGAACACCCCGTGGAAAACACCCAGCTGCAGAACGAGAAGCTGTACCTGTACTACCTGCAGAATGGGCGGGATATGTACGTGGACCAGGAACTGGACATCAACCGGCTGTCCGACTACGATGTGGACgccATCGTGCCTCAGAGCTTTCTGAAGGACGACTCCATCGACAACAAGGTGCTGACCAGAAGCGACAAGAACCGGGGCAAGAGCGACAACGTGCCCTCCGAAGAGGTCGTGAAGAAGATGAAGAACTACTGGCGGCAGCTGCTGAACGCCAAGCTGATTACCCAGAGAAAGTTCGACAATCTGACCAAGGCCGAGAGAGGCGGCCTGAGCGAACTGGATAAGGCCGGCTTCATCAAGAGACAGCTGGTGGAAACCCGGCAGATCACAAAGCACGTGGCACAGATCCTGGACTCCCGGATGAACACTAAGTACGACGAGAATGACAAGCTGATCCGGGAAGTGAAAGTGATCACCCTGAAGTCCAAGCTGGTGTCCGATTTCCGGAAGGATTTCCAGTTTTACAAAGTGCGCGAGATCAACAACTACCACCACGCCCACGACGCCTACCTGAACGCCGTCGTGGGAACCGCCCTGATCAAAAAGTACCCTAAGCTGGAAAGCGAGTTCGTGTACGGCGACTACAAGGTGTACGACGTGCGGAAGATGATCGCCAAGAGCGAGCAGGAAATCGGCAAGGCTACCGCCAAGTACTTCTTCTACAGCAACATCATGAACTTTTTCAAGACCGAGATTACCCTGGCCAACGGCGAGATCCGGAAGCGGCCTCTGATCGAGACAAACGGCGAAACCGGGGAGATCGTGTGGGATAAGGGCCGGGATTTTGCCACCGTGCGGAAAGTGCTGAGCATGCCCCAAGTGAATATCGTGAAAAAGACCGAGGTGCAGACAGGCGGCTTCAGCAAAGAGTCTATCCTGCCCAAGAGGAACAGCGATAAGCTGATCGCCAGAAAGAAGGACTGGGACCCTAAGAAGTACGGCGGCTTCGACAGCCCCACCGTGGCCTATTCTGTGCTGGTGGTGGCCAAAGTGGAAAAGGGCAAGTCCAAGAAACTGAAGAGTGTGAAAGAGCTGCTGGGGATCACCATCATGGAAAGAAGCAGCTTCGAGAAGAATCCCATCGACTTTCTGGAAGCCAAGGGCTACAAAGAAGTGAAAAAGGACCTGATCATCAAGCTGCCTAAGTACTCCCTGTTCGAGCTGGAAAACGGCCGGAAGAGAATGCTGGCCTCTGCCGGCGAACTGCAGAAGGGAAACGAACTGGCCCTGCCCTCCAAATATGTGAACTTCCTGTACCTGGCCAGCCACTATGAGAAGCTGAAGGGCTCCCCCGAGGATAATGAGCAGAAACAGCTGTTTGTGGAACAGCACAAGCACTACCTGGACGAGATCATCGAGCAGATCAGCGAGTTCTCCAAGAGAGTGATCCTGGCCGACGCTAATCTGGACAAAGTGCTGTCCGCCTACAACAAGCACCGGGATAAGCCCATCAGAGAGCAGGCCGAGAATATCATCCACCTGTTTACCCTGACCAATCTGGGAGCCCCTGCCGCCTTCAAGTACTTTGACACCACCATCGACCGGAAGAGGTACACCAGCACCAAAGAGGTGCTGGACGCCACCCTGATCCACCAGAGCATCACCGGCCTGTACGAGACACGGATCGACCTGTCTCAGCTGGGAGGCGACAAAAGGCCGGCGGCCACGAAAAAGGCCGGCCAGGCAAAAAAGAAAAAG**GGTTCCGGACGGGCT**GACGCATTGGACGATTTTGATCTGGATATGCTGGGAAGTGACGCCCTCGATGATTTTGACCTTGACATGCTTGGTTCGGATGCCCTTGATGACTTTGACCTCGACATGCTCGGCAGTGACGCCCTTGATGATTTCGACCTGGACATGCTG**ATTAACTCTCGCTAA

**3xFLAG-SV40 NLS-dCas9- HA-2x SV40 NLS-4x 22sTag-SV40 NLS**

**GACTATAAGGACCACGACGGAGACTACAAGGATCATGATATTGATTACAAAGACGATGA**CGATAAGATGGCC**CCAAAGAAGAAGCGGAAGGTC**GGTATCCACGGAGTCCCAGCAGCC**GACAAGAAGTACAGCATCGGCCTGGCCATCGGCACCAACTCTGTGGGCTGGGCCGTGATCACCGACGAGTACAAGGTGCCCAGCAAGAAATTCAAGGTGCTGGGCAACACCGACCGGCACAGCATCAAGAAGAACCTGATCGGAGCCCTGCTGTTCGACAGCGGCGAAACAGCCGAGGCCACCCGGCTGAAGAGAACCGCCAGAAGAAGATACACCAGACGGAAGAACCGGATCTGCTATCTGCAAGAGATCTTCAGCAACGAGATGGCCAAGGTGGACGACAGCTTCTTCCACAGACTGGAAGAGTCCTTCCTGGTGGAAGAGGATAAGAAGCACGAGCGGCACCCCATCTTCGGCAACATCGTGGACGAGGTGGCCTACCACGAGAAGTACCCCACCATCTACCACCTGAGAAAGAAACTGGTGGACAGCACCGACAAGGCCGACCTGCGGCTGATCTATCTGGCCCTGGCCCACATGATCAAGTTCCGGGGCCACTTCCTGATCGAGGGCGACCTGAACCCCGACAACAGCGACGTGGACAAGCTGTTCATCCAGCTGGTGCAGACCTACAACCAGCTGTTCGAGGAAAACCCCATCAACGCCAGCGGCGTGGACGCCAAGGCCATCCTGTCTGCCAGACTGAGCAAGAGCAGACGGCTGGAAAATCTGATCGCCCAGCTGCCCGGCGAGAAGAAGAATGGCCTGTTCGGAAACCTGATTGCCCTGAGCCTGGGCCTGACCCCCAACTTCAAGAGCAACTTCGACCTGGCCGAGGATGCCAAACTGCAGCTGAGCAAGGACACCTACGACGACGACCTGGACAACCTGCTGGCCCAGATCGGCGACCAGTACGCCGACCTGTTTCTGGCCGCCAAGAACCTGTCCGACGCCATCCTGCTGAGCGACATCCTGAGAGTGAACACCGAGATCACCAAGGCCCCCCTGAGCGCCTCTATGATCAAGAGATACGACGAGCACCACCAGGACCTGACCCTGCTGAAAGCTCTCGTGCGGCAGCAGCTGCCTGAGAAGTACAAAGAGATTTTCTTCGACCAGAGCAAGAACGGCTACGCCGGCTACATTGACGGCGGAGCCAGCCAGGAAGAGTTCTACAAGTTCATCAAGCCCATCCTGGAAAAGATGGACGGCACCGAGGAACTGCTCGTGAAGCTGAACAGAGAGGACCTGCTGCGGAAGCAGCGGACCTTCGACAACGGCAGCATCCCCCACCAGATCCACCTGGGAGAGCTGCACGCCATTCTGCGGCGGCAGGAAGATTTTTACCCATTCCTGAAGGACAACCGGGAAAAGATCGAGAAGATCCTGACCTTCCGCATCCCCTACTACGTGGGCCCTCTGGCCAGGGGAAACAGCAGATTCGCCTGGATGACCAGAAAGAGCGAGGAAACCATCACCCCCTGGAACTTCGAGGAAGTGGTGGACAAGGGCGCTTCCGCCCAGAGCTTCATCGAGCGGATGACCAACTTCGATAAGAACCTGCCCAACGAGAAGGTGCTGCCCAAGCACAGCCTGCTGTACGAGTACTTCACCGTGTATAACGAGCTGACCAAAGTGAAATACGTGACCGAGGGAATGAGAAAGCCCGCCTTCCTGAGCGGCGAGCAGAAAAAGGCCATCGTGGACCTGCTGTTCAAGACCAACCGGAAAGTGACCGTGAAGCAGCTGAAAGAGGACTACTTCAAGAAAATCGAGTGCTTCGACTCCGTGGAAATCTCCGGCGTGGAAGATCGGTTCAACGCCTCCCTGGGCACATACCACGATCTGCTGAAAATTATCAAGGACAAGGACTTCCTGGACAATGAGGAAAACGAGGACATTCTGGAAGATATCGTGCTGACCCTGACACTGTTTGAGGACAGAGAGATGATCGAGGAACGGCTGAAAACCTATGCCCACCTGTTCGACGACAAAGTGATGAAGCAGCTGAAGCGGCGGAGATACACCGGCTGGGGCAGGCTGAGCCGGAAGCTGATCAACGGCATCCGGGACAAGCAGTCCGGCAAGACAATCCTGGATTTCCTGAAGTCCGACGGCTTCGCCAACAGAAACTTCATGCAGCTGATCCACGACGACAGCCTGACCTTTAAAGAGGACATCCAGAAAGCCCAGGTGTCCGGCCAGGGCGATAGCCTGCACGAGCACATTGCCAATCTGGCCGGCAGCCCCGCCATTAAGAAGGGCATCCTGCAGACAGTGAAGGTGGTGGACGAGCTCGTGAAAGTGATGGGCCGGCACAAGCCCGAGAACATCGTGATCGAAATGGCCAGAGAGAACCAGACCACCCAGAAGGGACAGAAGAACAGCCGCGAGAGAATGAAGCGGATCGAAGAGGGCATCAAAGAGCTGGGCAGCCAGATCCTGAAAGAACACCCCGTGGAAAACACCCAGCTGCAGAACGAGAAGCTGTACCTGTACTACCTGCAGAATGGGCGGGATATGTACGTGGACCAGGAACTGGACATCAACCGGCTGTCCGACTACGATGTGGACgccATCGTGCCTCAGAGCTTTCTGAAGGACGACTCCATCGACAACAAGGTGCTGACCAGAAGCGACAAGAACCGGGGCAAGAGCGACAACGTGCCCTCCGAAGAGGTCGTGAAGAAGATGAAGAACTACTGGCGGCAGCTGCTGAACGCCAAGCTGATTACCCAGAGAAAGTTCGACAATCTGACCAAGGCCGAGAGAGGCGGCCTGAGCGAACTGGATAAGGCCGGCTTCATCAAGAGACAGCTGGTGGAAACCCGGCAGATCACAAAGCACGTGGCACAGATCCTGGACTCCCGGATGAACACTAAGTACGACGAGAATGACAAGCTGATCCGGGAAGTGAAAGTGATCACCCTGAAGTCCAAGCTGGTGTCCGATTTCCGGAAGGATTTCCAGTTTTACAAAGTGCGCGAGATCAACAACTACCACCACGCCCACGACGCCTACCTGAACGCCGTCGTGGGAACCGCCCTGATCAAAAAGTACCCTAAGCTGGAAAGCGAGTTCGTGTACGGCGACTACAAGGTGTACGACGTGCGGAAGATGATCGCCAAGAGCGAGCAGGAAATCGGCAAGGCTACCGCCAAGTACTTCTTCTACAGCAACATCATGAACTTTTTCAAGACCGAGATTACCCTGGCCAACGGCGAGATCCGGAAGCGGCCTCTGATCGAGACAAACGGCGAAACCGGGGAGATCGTGTGGGATAAGGGCCGGGATTTTGCCACCGTGCGGAAAGTGCTGAGCATGCCCCAAGTGAATATCGTGAAAAAGACCGAGGTGCAGACAGGCGGCTTCAGCAAAGAGTCTATCCTGCCCAAGAGGAACAGCGATAAGCTGATCGCCAGAAAGAAGGACTGGGACCCTAAGAAGTACGGCGGCTTCGACAGCCCCACCGTGGCCTATTCTGTGCTGGTGGTGGCCAAAGTGGAAAAGGGCAAGTCCAAGAAACTGAAGAGTGTGAAAGAGCTGCTGGGGATCACCATCATGGAAAGAAGCAGCTTCGAGAAGAATCCCATCGACTTTCTGGAAGCCAAGGGCTACAAAGAAGTGAAAAAGGACCTGATCATCAAGCTGCCTAAGTACTCCCTGTTCGAGCTGGAAAACGGCCGGAAGAGAATGCTGGCCTCTGCCGGCGAACTGCAGAAGGGAAACGAACTGGCCCTGCCCTCCAAATATGTGAACTTCCTGTACCTGGCCAGCCACTATGAGAAGCTGAAGGGCTCCCCCGAGGATAATGAGCAGAAACAGCTGTTTGTGGAACAGCACAAGCACTACCTGGACGAGATCATCGAGCAGATCAGCGAGTTCTCCAAGAGAGTGATCCTGGCCGACGCTAATCTGGACAAAGTGCTGTCCGCCTACAACAAGCACCGGGATAAGCCCATCAGAGAGCAGGCCGAGAATATCATCCACCTGTTTACCCTGACCAATCTGGGAGCCCCTGCCGCCTTCAAGTACTTTGACACCACCATCGACCGGAAGAGGTACACCAGCACCAAAGAGGTGCTGGACGCCACCCTGATCCACCAGAGCATCACCGGCCTGTACGAGACACGGATCGACCTGTCTCAGCTGGGAGGCGAC**GCC**TATCCCTATGACGTGCCCGATTATGCC**AGCCTGGGCAGCGGCTCC**CCCAAGAAAAAACGCAAGGTG**GAAGAT**CCTAAGAAAAAGCGGAAAGTG**GACGGCATTGGTAGTGGGAGCAACGGCAGCAGCGGATCCAACGGTCCGACTGACGCCGCGGAA**GAAGAGCTCCTTAGTAAGAACTATCATCTGGAAAATGAGGTAGCGCGCTTAAAGAAA**GGGTCGGGAAGTGGCGGCAGCGGAAGTGGGAGTGGAGGGAGCGGTTCTGGCGGTTCCGGCAGTGGA**GAGGAGTTGCTGTCTAAGAACTACCACTTAGAAAACGAAGTCGCACGGCTAAAAAAA**GGTTCTGGCTCCGGAGGCAGTGGTTCTGGAAGCGGTGGCAGCGGGTCAGGTGGAAGCGGATCAGGT**GAGGAATTGCTTTCCAAAAACTACCACCTTGAGAATGAGGTGGCCAGGTTAAAGAAG**GGCAGCGGCTCGGGGGGTAGTGGATCGGGGAGTGGCGGGTCAGGAAGCGGTGGTAGCGGAAGCGGG**GAGGAGCTGCTCTCGAAGAATTACCATTTGGAGAACGAAGTGGCGAGACTAAAGAAG**GGAAGCGGCAGTGGTGGATCTGGGTCTGGTTCAGGTGGGAGTGGGAGCGGTGGCTCAGGCTCAGGCACCGCGGTAAACATAGGTGGTGGAACCGGTCCGATGGATCTACAGCGGCCGCAAGGTGGAGGTGGA**CCCAAGAAGAAGCGCAAGGTGTAA**

**3xFLAG-SV40 NLS-dCas9- HA-2x SV40 NLS-8x 22sTag-SV40 NLS**

**GACTATAAGGACCACGACGGAGACTACAAGGATCATGATATTGATTACAAAGACGATGA**CGATAAGATGGCC**CCAAAGAAGAAGCGGAAGGTC**GGTATCCACGGAGTCCCAGCAGCC**GACAAGAAGTACAGCATCGGCCTGGCCATCGGCACCAACTCTGTGGGCTGGGCCGTGATCACCGACGAGTACAAGGTGCCCAGCAAGAAATTCAAGGTGCTGGGCAACACCGACCGGCACAGCATCAAGAAGAACCTGATCGGAGCCCTGCTGTTCGACAGCGGCGAAACAGCCGAGGCCACCCGGCTGAAGAGAACCGCCAGAAGAAGATACACCAGACGGAAGAACCGGATCTGCTATCTGCAAGAGATCTTCAGCAACGAGATGGCCAAGGTGGACGACAGCTTCTTCCACAGACTGGAAGAGTCCTTCCTGGTGGAAGAGGATAAGAAGCACGAGCGGCACCCCATCTTCGGCAACATCGTGGACGAGGTGGCCTACCACGAGAAGTACCCCACCATCTACCACCTGAGAAAGAAACTGGTGGACAGCACCGACAAGGCCGACCTGCGGCTGATCTATCTGGCCCTGGCCCACATGATCAAGTTCCGGGGCCACTTCCTGATCGAGGGCGACCTGAACCCCGACAACAGCGACGTGGACAAGCTGTTCATCCAGCTGGTGCAGACCTACAACCAGCTGTTCGAGGAAAACCCCATCAACGCCAGCGGCGTGGACGCCAAGGCCATCCTGTCTGCCAGACTGAGCAAGAGCAGACGGCTGGAAAATCTGATCGCCCAGCTGCCCGGCGAGAAGAAGAATGGCCTGTTCGGAAACCTGATTGCCCTGAGCCTGGGCCTGACCCCCAACTTCAAGAGCAACTTCGACCTGGCCGAGGATGCCAAACTGCAGCTGAGCAAGGACACCTACGACGACGACCTGGACAACCTGCTGGCCCAGATCGGCGACCAGTACGCCGACCTGTTTCTGGCCGCCAAGAACCTGTCCGACGCCATCCTGCTGAGCGACATCCTGAGAGTGAACACCGAGATCACCAAGGCCCCCCTGAGCGCCTCTATGATCAAGAGATACGACGAGCACCACCAGGACCTGACCCTGCTGAAAGCTCTCGTGCGGCAGCAGCTGCCTGAGAAGTACAAAGAGATTTTCTTCGACCAGAGCAAGAACGGCTACGCCGGCTACATTGACGGCGGAGCCAGCCAGGAAGAGTTCTACAAGTTCATCAAGCCCATCCTGGAAAAGATGGACGGCACCGAGGAACTGCTCGTGAAGCTGAACAGAGAGGACCTGCTGCGGAAGCAGCGGACCTTCGACAACGGCAGCATCCCCCACCAGATCCACCTGGGAGAGCTGCACGCCATTCTGCGGCGGCAGGAAGATTTTTACCCATTCCTGAAGGACAACCGGGAAAAGATCGAGAAGATCCTGACCTTCCGCATCCCCTACTACGTGGGCCCTCTGGCCAGGGGAAACAGCAGATTCGCCTGGATGACCAGAAAGAGCGAGGAAACCATCACCCCCTGGAACTTCGAGGAAGTGGTGGACAAGGGCGCTTCCGCCCAGAGCTTCATCGAGCGGATGACCAACTTCGATAAGAACCTGCCCAACGAGAAGGTGCTGCCCAAGCACAGCCTGCTGTACGAGTACTTCACCGTGTATAACGAGCTGACCAAAGTGAAATACGTGACCGAGGGAATGAGAAAGCCCGCCTTCCTGAGCGGCGAGCAGAAAAAGGCCATCGTGGACCTGCTGTTCAAGACCAACCGGAAAGTGACCGTGAAGCAGCTGAAAGAGGACTACTTCAAGAAAATCGAGTGCTTCGACTCCGTGGAAATCTCCGGCGTGGAAGATCGGTTCAACGCCTCCCTGGGCACATACCACGATCTGCTGAAAATTATCAAGGACAAGGACTTCCTGGACAATGAGGAAAACGAGGACATTCTGGAAGATATCGTGCTGACCCTGACACTGTTTGAGGACAGAGAGATGATCGAGGAACGGCTGAAAACCTATGCCCACCTGTTCGACGACAAAGTGATGAAGCAGCTGAAGCGGCGGAGATACACCGGCTGGGGCAGGCTGAGCCGGAAGCTGATCAACGGCATCCGGGACAAGCAGTCCGGCAAGACAATCCTGGATTTCCTGAAGTCCGACGGCTTCGCCAACAGAAACTTCATGCAGCTGATCCACGACGACAGCCTGACCTTTAAAGAGGACATCCAGAAAGCCCAGGTGTCCGGCCAGGGCGATAGCCTGCACGAGCACATTGCCAATCTGGCCGGCAGCCCCGCCATTAAGAAGGGCATCCTGCAGACAGTGAAGGTGGTGGACGAGCTCGTGAAAGTGATGGGCCGGCACAAGCCCGAGAACATCGTGATCGAAATGGCCAGAGAGAACCAGACCACCCAGAAGGGACAGAAGAACAGCCGCGAGAGAATGAAGCGGATCGAAGAGGGCATCAAAGAGCTGGGCAGCCAGATCCTGAAAGAACACCCCGTGGAAAACACCCAGCTGCAGAACGAGAAGCTGTACCTGTACTACCTGCAGAATGGGCGGGATATGTACGTGGACCAGGAACTGGACATCAACCGGCTGTCCGACTACGATGTGGACgccATCGTGCCTCAGAGCTTTCTGAAGGACGACTCCATCGACAACAAGGTGCTGACCAGAAGCGACAAGAACCGGGGCAAGAGCGACAACGTGCCCTCCGAAGAGGTCGTGAAGAAGATGAAGAACTACTGGCGGCAGCTGCTGAACGCCAAGCTGATTACCCAGAGAAAGTTCGACAATCTGACCAAGGCCGAGAGAGGCGGCCTGAGCGAACTGGATAAGGCCGGCTTCATCAAGAGACAGCTGGTGGAAACCCGGCAGATCACAAAGCACGTGGCACAGATCCTGGACTCCCGGATGAACACTAAGTACGACGAGAATGACAAGCTGATCCGGGAAGTGAAAGTGATCACCCTGAAGTCCAAGCTGGTGTCCGATTTCCGGAAGGATTTCCAGTTTTACAAAGTGCGCGAGATCAACAACTACCACCACGCCCACGACGCCTACCTGAACGCCGTCGTGGGAACCGCCCTGATCAAAAAGTACCCTAAGCTGGAAAGCGAGTTCGTGTACGGCGACTACAAGGTGTACGACGTGCGGAAGATGATCGCCAAGAGCGAGCAGGAAATCGGCAAGGCTACCGCCAAGTACTTCTTCTACAGCAACATCATGAACTTTTTCAAGACCGAGATTACCCTGGCCAACGGCGAGATCCGGAAGCGGCCTCTGATCGAGACAAACGGCGAAACCGGGGAGATCGTGTGGGATAAGGGCCGGGATTTTGCCACCGTGCGGAAAGTGCTGAGCATGCCCCAAGTGAATATCGTGAAAAAGACCGAGGTGCAGACAGGCGGCTTCAGCAAAGAGTCTATCCTGCCCAAGAGGAACAGCGATAAGCTGATCGCCAGAAAGAAGGACTGGGACCCTAAGAAGTACGGCGGCTTCGACAGCCCCACCGTGGCCTATTCTGTGCTGGTGGTGGCCAAAGTGGAAAAGGGCAAGTCCAAGAAACTGAAGAGTGTGAAAGAGCTGCTGGGGATCACCATCATGGAAAGAAGCAGCTTCGAGAAGAATCCCATCGACTTTCTGGAAGCCAAGGGCTACAAAGAAGTGAAAAAGGACCTGATCATCAAGCTGCCTAAGTACTCCCTGTTCGAGCTGGAAAACGGCCGGAAGAGAATGCTGGCCTCTGCCGGCGAACTGCAGAAGGGAAACGAACTGGCCCTGCCCTCCAAATATGTGAACTTCCTGTACCTGGCCAGCCACTATGAGAAGCTGAAGGGCTCCCCCGAGGATAATGAGCAGAAACAGCTGTTTGTGGAACAGCACAAGCACTACCTGGACGAGATCATCGAGCAGATCAGCGAGTTCTCCAAGAGAGTGATCCTGGCCGACGCTAATCTGGACAAAGTGCTGTCCGCCTACAACAAGCACCGGGATAAGCCCATCAGAGAGCAGGCCGAGAATATCATCCACCTGTTTACCCTGACCAATCTGGGAGCCCCTGCCGCCTTCAAGTACTTTGACACCACCATCGACCGGAAGAGGTACACCAGCACCAAAGAGGTGCTGGACGCCACCCTGATCCACCAGAGCATCACCGGCCTGTACGAGACACGGATCGACCTGTCTCAGCTGGGAGGCGAC**GCC**TATCCCTATGACGTGCCCGATTATGCC**AGCCTGGGCAGCGGCTCC**CCCAAGAAAAAACGCAAGGTG**GAAGAT**CCTAAGAAAAAGCGGAAAGTG**GACGGCATTGGTAGTGGGAGCAACGGCAGCAGCGGATCCAACGGTCCGACTGACGCCGCGGAA**GAAGAGCTCCTTAGTAAGAACTATCATCTGGAAAATGAGGTAGCGCGCTTAAAGAAA**GGGTCGGGAAGTGGCGGCAGCGGAAGTGGGAGTGGAGGGAGCGGTTCTGGCGGTTCCGGCAGTGGA**GAGGAGTTGCTGTCTAAGAACTACCACTTAGAAAACGAAGTCGCACGGCTAAAAAAA**GGTTCTGGCTCCGGAGGCAGTGGTTCTGGAAGCGGTGGCAGCGGGTCAGGTGGAAGCGGATCAGGT**GAGGAATTGCTTTCCAAAAACTACCACCTTGAGAATGAGGTGGCCAGGTTAAAGAAG**GGCAGCGGCTCGGGGGGTAGTGGATCGGGGAGTGGCGGGTCAGGAAGCGGTGGTAGCGGAAGCGGG**GAGGAGCTGCTCTCGAAGAATTACCATTTGGAGAACGAAGTGGCGAGACTAAAGAAG**GGAAGCGGCAGTGGTGGATCTGGGTCTGGTTCAGGTGGGAGTGGGAGCGGTGGCTCAGGGTCTGGG**GAAGAGCTCCTTAGTAAGAACTATCATCTGGAAAATGAGGTAGCGCGCTTAAAGAAA**GGGTCGGGAAGTGGCGGCAGCGGAAGTGGGAGTGGAGGGAGCGGTTCTGGCGGTTCCGGCAGTGGA**GAGGAGTTGCTGTCTAAGAACTACCACTTAGAAAACGAAGTCGCACGGCTAAAAAAA**GGTTCTGGCTCCGGAGGCAGTGGTTCTGGAAGCGGTGGCAGCGGGTCAGGTGGAAGCGGATCAGGT**GAGGAATTGCTTTCCAAAAACTACCACCTTGAGAATGAGGTGGCCAGGTTAAAGAAG**GGCAGCGGCTCGGGGGGTAGTGGATCGGGGAGTGGCGGGTCAGGAAGCGGTGGTAGCGGAAGCGGG**GAGGAGCTGCTCTCGAAGAATTACCATTTGGAGAACGAAGTGGCGAGACTAAAGAAG**GGAAGCGGCAGTGGTGGATCTGGGTCTGGTTCAGGTGGGAGTGGGAGCGGTGGCTCAGGCTCAGGCACCGCGGtAAACATAGGTGGTGGAACCGGTCCGATGGATCTACAGCGGCCGCAAGGTGGAGGTGGA**CCCAAGAAGAAGCGCAAGGTG**TAA

**MS2-SV40 NLS-HA-2x SV40 NLS-4x 22sTag-SV40 NLS**

**GCTTCAAACTTTACTCAGTTCGTGCTCGTGGACAATGGTGGGACAGGGGATGTGACAGTGGCTCCTTCTAATTTCGCTAATGGGGTGGCAGAGTGGATCAGCTCCAACTCACGGAGCCAGGCCTACAAGGTGACATGCAGCGTCAGGCAGTCTAGTGCCCAGAAGAGAAAGTATACCATCAAGGTGGAGGTCCCCAAAGTGGCTACCCAGACAGTGGGCGGAGTCGAACTGCCTGTCGCCGCTTGGAGGTCCTACCTGAACATGGAGCTCACTATCCCAATTTTCGCTACCAATTCTGACTGTGAACTCATCGTGAAGGCAATGCAGGGGCTCCTCAAAGACGGTAATCCTATCCCTTCCGCCATCGCCGCTAACTCAGGTATCTAC**AGCGCTGGAGGAGGTGGAAGCGGAGGAGGAGGAAGCGGAGGAGGAGGTAGCGGA**CCTAAGAAAAAGAGGAAGGTG**GCGGCCGCTGGATCCGCC**TATCCCTATGACGTGCCCGATTATGCC**AGCCTGGGCAGCGGCTCC**CCCAAGAAAAAACGCAAGGTG**GAAGAT**CCTAAGAAAAAGCGGAAAGTG**GACGGCATTGGTAGTGGGAGCAACGGCAGCAGCGGATCCAACGGTCCGACTGACGCCGCGGAA**GAAGAGCTCCTTAGTAAGAACTATCATCTGGAAAATGAGGTAGCGCGCTTAAAGAAA**GGGTCGGGAAGTGGCGGCAGCGGAAGTGGGAGTGGAGGGAGCGGTTCTGGCGGTTCCGGCAGTGGA**GAGGAGTTGCTGTCTAAGAACTACCACTTAGAAAACGAAGTCGCACGGCTAAAAAAA**GGTTCTGGCTCCGGAGGCAGTGGTTCTGGAAGCGGTGGCAGCGGGTCAGGTGGAAGCGGATCAGGT**GAGGAATTGCTTTCCAAAAACTACCACCTTGAGAATGAGGTGGCCAGGTTAAAGAAG**GGCAGCGGCTCGGGGGGTAGTGGATCGGGGAGTGGCGGGTCAGGAAGCGGTGGTAGCGGAAGCGGG**GAGGAGCTGCTCTCGAAGAATTACCATTTGGAGAACGAAGTGGCGAGACTAAAGAAG**GGAAGCGGCAGTGGTGGATCTGGGTCTGGTTCAGGTGGGAGTGGGAGCGGTGGCTCAGGCTCAGGCACCGCGGTAAACATAGGTGGTGGAACCGGTCCGATGGATCTACAGCGGCCGCAAGGTGGAGGTGGA**CCCAAGAAGAAGCGCAAGGTG**TAA

**MS2-SV40 NLS-HA-2x SV40 NLS-8x 22sTag-SV40 NLS**

**GCTTCAAACTTTACTCAGTTCGTGCTCGTGGACAATGGTGGGACAGGGGATGTGACAGTGGCTCCTTCTAATTTCGCTAATGGGGTGGCAGAGTGGATCAGCTCCAACTCACGGAGCCAGGCCTACAAGGTGACATGCAGCGTCAGGCAGTCTAGTGCCCAGAAGAGAAAGTATACCATCAAGGTGGAGGTCCCCAAAGTGGCTACCCAGACAGTGGGCGGAGTCGAACTGCCTGTCGCCGCTTGGAGGTCCTACCTGAACATGGAGCTCACTATCCCAATTTTCGCTACCAATTCTGACTGTGAACTCATCGTGAAGGCAATGCAGGGGCTCCTCAAAGACGGTAATCCTATCCCTTCCGCCATCGCCGCTAACTCAGGTATCTAC**AGCGCTGGAGGAGGTGGAAGCGGAGGAGGAGGAAGCGGAGGAGGAGGTAGCGGA**CCTAAGAAAAAGAGGAAGGTG**GCGGCCGCTGGATCCGCC**TATCCCTATGACGTGCCCGATTATGCC**AGCCTGGGCAGCGGCTCC**CCCAAGAAAAAACGCAAGGTG**GAAGAT**CCTAAGAAAAAGCGGAAAGTG**GACGGCATTGGTAGTGGGAGCAACGGCAGCAGCGGATCCAACGGTCCGACTGACGCCGCGGAA**GAAGAGCTCCTTAGTAAGAACTATCATCTGGAAAATGAGGTAGCGCGCTTAAAGAAA**GGGTCGGGAAGTGGCGGCAGCGGAAGTGGGAGTGGAGGGAGCGGTTCTGGCGGTTCCGGCAGTGGA**GAGGAGTTGCTGTCTAAGAACTACCACTTAGAAAACGAAGTCGCACGGCTAAAAAAA**GGTTCTGGCTCCGGAGGCAGTGGTTCTGGAAGCGGTGGCAGCGGGTCAGGTGGAAGCGGATCAGGT**GAGGAATTGCTTTCCAAAAACTACCACCTTGAGAATGAGGTGGCCAGGTTAAAGAAG**GGCAGCGGCTCGGGGGGTAGTGGATCGGGGAGTGGCGGGTCAGGAAGCGGTGGTAGCGGAAGCGGG**GAGGAGCTGCTCTCGAAGAATTACCATTTGGAGAACGAAGTGGCGAGACTAAAGAAG**GGAAGCGGCAGTGGTGGATCTGGGTCTGGTTCAGGTGGGAGTGGGAGCGGTGGCTCAGGGTCTGGG**GAAGAGCTCCTTAGTAAGAACTATCATCTGGAAAATGAGGTAGCGCGCTTAAAGAAA**GGGTCGGGAAGTGGCGGCAGCGGAAGTGGGAGTGGAGGGAGCGGTTCTGGCGGTTCCGGCAGTGGA**GAGGAGTTGCTGTCTAAGAACTACCACTTAGAAAACGAAGTCGCACGGCTAAAAAAA**GGTTCTGGCTCCGGAGGCAGTGGTTCTGGAAGCGGTGGCAGCGGGTCAGGTGGAAGCGGATCAGGT**GAGGAATTGCTTTCCAAAAACTACCACCTTGAGAATGAGGTGGCCAGGTTAAAGAAG**GGCAGCGGCTCGGGGGGTAGTGGATCGGGGAGTGGCGGGTCAGGAAGCGGTGGTAGCGGAAGCGGG**GAGGAGCTGCTCTCGAAGAATTACCATTTGGAGAACGAAGTGGCGAGACTAAAGAAG**GGAAGCGGCAGTGGTGGATCTGGGTCTGGTTCAGGTGGGAGTGGGAGCGGTGGCTCAGGCTCAGGCACCGCGGtAAACATAGGTGGTGGAACCGGTCCGATGGATCTACAGCGGCCGCAAGGTGGAGGTGGA**CCCAAGAAGAAGCGCAAGGTG**TAA

**MS2-SV40 NLS-HA-2x SV40 NLS-16x 22sTag-SV40 NLS**

**GCTTCAAACTTTACTCAGTTCGTGCTCGTGGACAATGGTGGGACAGGGGATGTGACAGTGGCTCCTTCTAATTTCGCTAATGGGGTGGCAGAGTGGATCAGCTCCAACTCACGGAGCCAGGCCTACAAGGTGACATGCAGCGTCAGGCAGTCTAGTGCCCAGAAGAGAAAGTATACCATCAAGGTGGAGGTCCCCAAAGTGGCTACCCAGACAGTGGGCGGAGTCGAACTGCCTGTCGCCGCTTGGAGGTCCTACCTGAACATGGAGCTCACTATCCCAATTTTCGCTACCAATTCTGACTGTGAACTCATCGTGAAGGCAATGCAGGGGCTCCTCAAAGACGGTAATCCTATCCCTTCCGCCATCGCCGCTAACTCAGGTATCTAC**AGCGCTGGAGGAGGTGGAAGCGGAGGAGGAGGAAGCGGAGGAGGAGGTAGCGGA**CCTAAGAAAAAGAGGAAGGTG**GCGGCCGCTGGATCCGCC**TATCCCTATGACGTGCCCGATTATGCC**AGCCTGGGCAGCGGCTCC**CCCAAGAAAAAACGCAAGGTG**GAAGAT**CCTAAGAAAAAGCGGAAAGTG**GACGGCATTGGTAGTGGGAGCAACGGCAGCAGCGGATCCAACGGTCCGACTGACGCCGCGGAA**GAAGAGCTCCTTAGTAAGAACTATCATCTGGAAAATGAGGTAGCGCGCTTAAAGAAA**GGGTCGGGAAGTGGCGGCAGCGGAAGTGGGAGTGGAGGGAGCGGTTCTGGCGGTTCCGGCAGTGGA**GAGGAGTTGCTGTCTAAGAACTACCACTTAGAAAACGAAGTCGCACGGCTAAAAAAA**GGTTCTGGCTCCGGAGGCAGTGGTTCTGGAAGCGGTGGCAGCGGGTCAGGTGGAAGCGGATCAGGT**GAGGAATTGCTTTCCAAAAACTACCACCTTGAGAATGAGGTGGCCAGGTTAAAGAAG**GGCAGCGGCTCGGGGGGTAGTGGATCGGGGAGTGGCGGGTCAGGAAGCGGTGGTAGCGGAAGCGGG**GAGGAGCTGCTCTCGAAGAATTACCATTTGGAGAACGAAGTGGCGAGACTAAAGAAG**GGAAGCGGCAGTGGTGGATCTGGGTCTGGTTCAGGTGGGAGTGGGAGCGGTGGCTCAGGGTCTGGG**GAAGAGCTCCTTAGTAAGAACTATCATCTGGAAAATGAGGTAGCGCGCTTAAAGAAA**GGGTCGGGAAGTGGCGGCAGCGGAAGTGGGAGTGGAGGGAGCGGTTCTGGCGGTTCCGGCAGTGGA**GAGGAGTTGCTGTCTAAGAACTACCACTTAGAAAACGAAGTCGCACGGCTAAAAAAA**GGTTCTGGCTCCGGAGGCAGTGGTTCTGGAAGCGGTGGCAGCGGGTCAGGTGGAAGCGGATCAGGT**GAGGAATTGCTTTCCAAAAACTACCACCTTGAGAATGAGGTGGCCAGGTTAAAGAAG**GGCAGCGGCTCGGGGGGTAGTGGATCGGGGAGTGGCGGGTCAGGAAGCGGTGGTAGCGGAAGCGGG**GAGGAGCTGCTCTCGAAGAATTACCATTTGGAGAACGAAGTGGCGAGACTAAAGAAG**GGAAGCGGCAGTGGTGGATCTGGGTCTGGTTCAGGTGGGAGTGGGAGCGGTGGCTCAGGCTCAGGCACCGCGGTAAACATAGGTGGTGGAACCGGTCCGATGGATCTACAGCGGCCGAGCAGCGGATCCAACGGTCCGACTGACGCCGCGGAA**GAAGAGCTCCTTAGTAAGAACTATCATCTGGAAAATGAGGTAGCGCGCTTAAAGAAA**GGGTCGGGAAGTGGCGGCAGCGGAAGTGGGAGTGGAGGGAGCGGTTCTGGCGGTTCCGGCAGTGGA**GAGGAGTTGCTGTCTAAGAACTACCACTTAGAAAACGAAGTCGCACGGCTAAAAAAA**GGTTCTGGCTCCGGAGGCAGTGGTTCTGGAAGCGGTGGCAGCGGGTCAGGTGGAAGCGGATCAGGT**GAGGAATTGCTTTCCAAAAACTACCACCTTGAGAATGAGGTGGCCAGGTTAAAGAAG**GGCAGCGGCTCGGGGGGTAGTGGATCGGGGAGTGGCGGGTCAGGAAGCGGTGGTAGCGGAAGCGGG**GAGGAGCTGCTCTCGAAGAATTACCATTTGGAGAACGAAGTGGCGAGACTAAAGAAG**GGAAGCGGCAGTGGTGGATCTGGGTCTGGTTCAGGTGGGAGTGGGAGCGGTGGCTCAGGGTCTGGGG**AAGAGCTCCTTAGTAAGAACTATCATCTGGAAAATGAGGTAGCGCGCTTAAAGAAA**GGGTCGGGAAGTGGCGGCAGCGGAAGTGGGAGTGGAGGGAGCGGTTCTGGCGGTTCCGGCAGTGGA**GAGGAGTTGCTGTCTAAGAACTACCACTTAGAAAACGAAGTCGCACGGCTAAAAAAA**GGTTCTGGCTCCGGAGGCAGTGGTTCTGGAAGCGGTGGCAGCGGGTCAGGTGGAAGCGGATCAGGT**GAGGAATTGCTTTCCAAAAACTACCACCTTGAGAATGAGGTGGCCAGGTTAAAGAAG**GGCAGCGGCTCGGGGGGTAGTGGATCGGGGAGTGGCGGGTCAGGAAGCGGTGGTAGCGGAAGCGGG**GAGGAGCTGCTCTCGAAGAATTACCATTTGGAGAACGAAGTGGCGAGACTAAAGAAG**GGAAGCGGCAGTGGTGGATCTGGGTCTGGTTCAGGTGGGAGTGGGAGCGGTGGCTCAGGCTCAGGCACCGCGGTAAACATAGGTGGTGGAACCGGTCCGATGGATCTACAGCGGCCGCAAGGTGGAGGTGGA**CCCAAGAAGAAGCGCAAGGTG**TAA

**MS2-SV40 NLS-HA-2x SV40 NLS-24x 22sTag-SV40 NLS**

**GCTTCAAACTTTACTCAGTTCGTGCTCGTGGACAATGGTGGGACAGGGGATGTGACAGTGGCTCCTTCTAATTTCGCTAATGGGGTGGCAGAGTGGATCAGCTCCAACTCACGGAGCCAGGCCTACAAGGTGACATGCAGCGTCAGGCAGTCTAGTGCCCAGAAGAGAAAGTATACCATCAAGGTGGAGGTCCCCAAAGTGGCTACCCAGACAGTGGGCGGAGTCGAACTGCCTGTCGCCGCTTGGAGGTCCTACCTGAACATGGAGCTCACTATCCCAATTTTCGCTACCAATTCTGACTGTGAACTCATCGTGAAGGCAATGCAGGGGCTCCTCAAAGACGGTAATCCTATCCCTTCCGCCATCGCCGCTAACTCAGGTATCTAC**AGCGCTGGAGGAGGTGGAAGCGGAGGAGGAGGAAGCGGAGGAGGAGGTAGCGGA**CCTAAGAAAAAGAGGAAGGTG**GCGGCCGCTGGATCCGCC**TATCCCTATGACGTGCCCGATTATGCC**AGCCTGGGCAGCGGCTCC**CCCAAGAAAAAACGCAAGGTG**GAAGAT**CCTAAGAAAAAGCGGAAAGTG**GACGGCATTGGTAGTGGGAGCAACGGCAGCAGCGGATCCAACGGTCCGACTGACGCCGCGGAA**GAAGAGCTCCTTAGTAAGAACTATCATCTGGAAAATGAGGTAGCGCGCTTAAAGAAA**GGGTCGGGAAGTGGCGGCAGCGGAAGTGGGAGTGGAGGGAGCGGTTCTGGCGGTTCCGGCAGTGGA**GAGGAGTTGCTGTCTAAGAACTACCACTTAGAAAACGAAGTCGCACGGCTAAAAAAA**GGTTCTGGCTCCGGAGGCAGTGGTTCTGGAAGCGGTGGCAGCGGGTCAGGTGGAAGCGGATCAGGT**GAGGAATTGCTTTCCAAAAACTACCACCTTGAGAATGAGGTGGCCAGGTTAAAGAAG**GGCAGCGGCTCGGGGGGTAGTGGATCGGGGAGTGGCGGGTCAGGAAGCGGTGGTAGCGGAAGCGGG**GAGGAGCTGCTCTCGAAGAATTACCATTTGGAGAACGAAGTGGCGAGACTAAAGAAG**GGAAGCGGCAGTGGTGGATCTGGGTCTGGTTCAGGTGGGAGTGGGAGCGGTGGCTCAGGGTCTGGG**GAAGAGCTCCTTAGTAAGAACTATCATCTGGAAAATGAGGTAGCGCGCTTAAAGAAA**GGGTCGGGAAGTGGCGGCAGCGGAAGTGGGAGTGGAGGGAGCGGTTCTGGCGGTTCCGGCAGTGGA**GAGGAGTTGCTGTCTAAGAACTACCACTTAGAAAACGAAGTCGCACGGCTAAAAAAA**GGTTCTGGCTCCGGAGGCAGTGGTTCTGGAAGCGGTGGCAGCGGGTCAGGTGGAAGCGGATCAGGT**GAGGAATTGCTTTCCAAAAACTACCACCTTGAGAATGAGGTGGCCAGGTTAAAGAAG**GGCAGCGGCTCGGGGGGTAGTGGATCGGGGAGTGGCGGGTCAGGAAGCGGTGGTAGCGGAAGCGGG**GAGGAGCTGCTCTCGAAGAATTACCATTTGGAGAACGAAGTGGCGAGACTAAAGAAG**GGAAGCGGCAGTGGTGGATCTGGGTCTGGTTCAGGTGGGAGTGGGAGCGGTGGCTCAGGCTCAGGCACCGCGGTAAACATAGGTGGTGGAACCGGTCCGATGGATCTACAGCGGCCGAGCAGCGGATCCAACGGTCCGACTGACGCCGCGGAA**GAAGAGCTCCTTAGTAAGAACTATCATCTGGAAAATGAGGTAGCGCGCTTAAAGAAA**GGGTCGGGAAGTGGCGGCAGCGGAAGTGGGAGTGGAGGGAGCGGTTCTGGCGGTTCCGGCAGTGGA**GAGGAGTTGCTGTCTAAGAACTACCACTTAGAAAACGAAGTCGCACGGCTAAAAAAA**GGTTCTGGCTCCGGAGGCAGTGGTTCTGGAAGCGGTGGCAGCGGGTCAGGTGGAAGCGGATCAGGT**GAGGAATTGCTTTCCAAAAACTACCACCTTGAGAATGAGGTGGCCAGGTTAAAGAAG**GGCAGCGGCTCGGGGGGTAGTGGATCGGGGAGTGGCGGGTCAGGAAGCGGTGGTAGCGGAAGCGGG**GAGGAGCTGCTCTCGAAGAATTACCATTTGGAGAACGAAGTGGCGAGACTAAAGAAG**GGAAGCGGCAGTGGTGGATCTGGGTCTGGTTCAGGTGGGAGTGGGAGCGGTGGCTCAGGGTCTGGGG**AAGAGCTCCTTAGTAAGAACTATCATCTGGAAAATGAGGTAGCGCGCTTAAAGAAA**GGGTCGGGAAGTGGCGGCAGCGGAAGTGGGAGTGGAGGGAGCGGTTCTGGCGGTTCCGGCAGTGGA**GAGGAGTTGCTGTCTAAGAACTACCACTTAGAAAACGAAGTCGCACGGCTAAAAAAA**GGTTCTGGCTCCGGAGGCAGTGGTTCTGGAAGCGGTGGCAGCGGGTCAGGTGGAAGCGGATCAGGT**GAGGAATTGCTTTCCAAAAACTACCACCTTGAGAATGAGGTGGCCAGGTTAAAGAAG**GGCAGCGGCTCGGGGGGTAGTGGATCGGGGAGTGGCGGGTCAGGAAGCGGTGGTAGCGGAAGCGGG**GAGGAGCTGCTCTCGAAGAATTACCATTTGGAGAACGAAGTGGCGAGACTAAAGAAG**GGAAGCGGCAGTGGTGGATCTGGGTCTGGTTCAGGTGGGAGTGGGAGCGGTGGCTCAGGCTCAGGCACCGCGGTAAACATAGGTGGTGGAACCGGTCCGATGGATCTACAGCGGCCGAGCAGCGGATCCAACGGTCCGACTGACGCCGCGGAA**GAAGAGCTCCTTAGTAAGAACTATCATCTGGAAAATGAGGTAGCGCGCTTAAAGAAA**GGGTCGGGAAGTGGCGGCAGCGGAAGTGGGAGTGGAGGGAGCGGTTCTGGCGGTTCCGGCAGTGGA**GAGGAGTTGCTGTCTAAGAACTACCACTTAGAAAACGAAGTCGCACGGCTAAAAAAA**GGTTCTGGCTCCGGAGGCAGTGGTTCTGGAAGCGGTGGCAGCGGGTCAGGTGGAAGCGGATCAGGT**GAGGAATTGCTTTCCAAAAACTACCACCTTGAGAATGAGGTGGCCAGGTTAAAGAAG**GGCAGCGGCTCGGGGGGTAGTGGATCGGGGAGTGGCGGGTCAGGAAGCGGTGGTAGCGGAAGCGGG**GAGGAGCTGCTCTCGAAGAATTACCATTTGGAGAACGAAGTGGCGAGACTAAAGAAG**GGAAGCGGCAGTGGTGGATCTGGGTCTGGTTCAGGTGGGAGTGGGAGCGGTGGCTCAGGGTCTGGG**GAAGAGCTCCTTAGTAAGAACTATCATCTGGAAAATGAGGTAGCGCGCTTAAAGAAA**GGGTCGGGAAGTGGCGGCAGCGGAAGTGGGAGTGGAGGGAGCGGTTCTGGCGGTTCCGGCAGTGGA**GAGGAGTTGCTGTCTAAGAACTACCACTTAGAAAACGAAGTCGCACGGCTAAAAAAA**GGTTCTGGCTCCGGAGGCAGTGGTTCTGGAAGCGGTGGCAGCGGGTCAGGTGGAAGCGGATCAGGT**GAGGAATTGCTTTCCAAAAACTACCACCTTGAGAATGAGGTGGCCAGGTTAAAGAAG**GGCAGCGGCTCGGGGGGTAGTGGATCGGGGAGTGGCGGGTCAGGAAGCGGTGGTAGCGGAAGCGGG**GAGGAGCTGCTCTCGAAGAATTACCATTTGGAGAACGAAGTGGCGAGACTAAAGAAG**GGAAGCGGCAGTGGTGGATCTGGGTCTGGTTCAGGTGGGAGTGGGAGCGGTGGCTCAGGCTCAGGCACCGCGGTAAACATAGGTGGTGGAACCGGTCCGATGGATCTACAGCGGCCGCAAGGTGGAGGTGGA**CCCAAGAAGAAGCGCAAGGTG**TAA

**scFv-sfGFP-p65-HSF1-GB1-Rex NLS**

**ATGGGCCCCGACATCGTGATGACCCAGAGCCCCAGCAGCCTGAGCGCCAGCGTGGGCGACCGCGTGACCATCACCTGCCGCAGCAGCACCGGCGCCGTGACCACCAGCAACTACGCCAGCTGGGTGCAGGAGAAGCCCGGCAAGCTGTTCAAGGGCCTGATCGGCGGCACCAACAACCGCGCCCCCGGCGTGCCCAGCCGCTTCAGCGGCAGCCTGATCGGCGACAAGGCCACCCTGACCATCAGCAGCCTGCAGCCCGAGGACTTCGCCACCTACTTCTGCGCCCTGTGGTACAGCAACCACTGGGTGTTCGGCCAGGGCACCAAGGTGGAGCTGAAGCGCGGCGGCGGCGGCAGCGGCGGCGGCGGCAGCGGCGGCGGCGGCAGCAGCGGCGGCGGCAGCGAGGTGAAGCTGCTGGAGAGCGGCGGCGGCCTGGTGCAGCCCGGCGGCAGCCTGAAGCTGAGCTGCGCCGTGAGCGGCTTCAGCCTGACCGACTACGGCGTGAACTGGGTGCGCCAGGCCCCCGGCCGCGGCCTGGAGTGGATCGGCGTGATCTGGGGCGACGGCATCACCGACTACAACAGCGCCCTGAAGGACCGCTTCATCATCAGCAAGGACAACGGCAAGAACACCGTGTACCTGCAGATGAGCAAGGTGCGCAGCGACGACACCGCCCTGTACTACTGCGTGACCGGCCTGTTCGACTACTGGGGCCAGGGCACCCTGGTGACCGTGAGCAGCTACCCATACGATGTTCCAGATTACGCTGGTGGAGGCGGAGGTTCTGGGGGAGGAGGTAGTGGCGGTGGTGGTTCAGGAGGCGGCGGAAGCTTGGATCCAGGTGGAGGTGGAAGCGGTAGCAAAGGAGAAGAACTTTTCACTGGAGTTGTCCCAATTCTTGTTGAATTAGATGGTGATGTTAATGGGCACAAATTTTCTGTCCGTGGAGAGGGTGAAGGTGATGCTACAAACGGAAAACTCACCCTTAAATTTATTTGCACTACTGGAAAACTACCTGTTCCGTGGCCAACACTTGTCACTACTCTGACCTATGGTGTTCAATGCTTTTCCCGTTATCCGGATCACATGAAACGGCATGACTTTTTCAAGAGTGCCATGCCCGAAGGTTATGTACAGGAACGCACTATATCTTTCAAAGATGACGGGACCTACAAGACGCGTGCTGAAGTCAAGTTTGAAGGTGATACCCTTGTTAATCGTATCGAGTTAAAGGGTATTGATTTTAAAGAAGATGGAAACATTCTTGGACACAAACTCGAGTACAACTTTAACTCACACAATGTATACATCACGGCAGACAAACAAAAGAATGGAATCAAAGCTAACTTCAAAATTCGCCACAACGTTGAAGATGGTTCCGTTCAACTAGCAGACCATTATCAACAAAATACTCCAATTGGCGATGGCCCTGTCCTTTTACCAGACAACCATTACCTGTCGACACAATCTGTCCTTTCGAAAGATCCCAACGAAAAGCGTGACCACATGGTCCTTCTTGAGTTTGTAACTGCTGCTGGGATTACACATGGCATGGATGAGCTCTACAAA**GGTGGAGGTCGGACCGGTGGCGGTGGCGGAGGG**CCTTCAGGGCAGATCAGCAACCAGGCCCTGGCTCTGGCCCCTAGCTCCGCTCCAGTGCTGGCCCAGACTATGGTGCCCTCTAGTGCTATGGTGCCTCTGGCCCAGCCACCTGCTCCAGCCCCTGTGCTGACCCCAGGACCACCCCAGTCACTGAGCGCTCCAGTGCCCAAGTCTACACAGGCCGGCGAGGGGACTCTGAGTGAAGCTCTGCTGCACCTGCAGTTCGACGCTGATGAGGACCTGGGAGCTCTGCTGGGGAACAGCACCGATCCCGGAGTGTTCACAGATCTGGCCTCCGTGGACAACTCTGAGTTTCAGCAGCTGCTGAATCAGGGCGTGTCCATGTCTCATAGTACAGCCGAACCAATGCTGATGGAGTACCCCGAAGCCATTACCCGGCTGGTGACCGGCAGCCAGCGGCCCCCCGACCCCGCTCCAACTCCCCTGGGAACCAGCGGCCTGCCTAATGGGCTGTCCGGAGATGAAGACTTCTCAAGCATCGCTGATATGGACTTTAGTGCCCTGCTGTCACAGATTTCCTCT**AGTGGGCAGGGAGGAGGTGGAAGC**GGCTTCAGCGTGGACACCAGTGCCCTGCTGGACCTGTTCAGCCCCTCGGTGACCGTGCCCGACATGAGCCTGCCTGACCTTGACAGCAGCCTGGCCAGTATCCAAGAGCTCCTGTCTCCCCAGGAGCCCCCCAGGCCTCCCGAGGCAGAGAACAGCAGCCCGGATTCAGGGAAGCAGCTGGTGCACTACACAGCGCAGCCGCTGTTCCTGCTGGACCCCGGCTCCGTGGACACCGGGAGCAACGACCTGCCGGTGCTGTTTGAGCTGGGAGAGGGCTCCTACTTCTCCGAAGGGGACGGCTTCGCCGAGGACCCCACCATCTCCCTGCTGACAGGCTCGGAGCCTCCCAAAGCCAAGGACCCCACTGTCTCC**GGTAGTGGAGGAGGATCTCGGACCGAAGAG**TACAAGCTTATCCTGAACGGTAAAACCCTGAAAGGTGAAACCACCACCGAAGCTGTTGACGCTGCTACCGCGGAAAAAGTTTTCAAACAGTACGCTAACGACAACGGTGTTGACGGTGAATGGACCTACGACGACGCTACCAAAACCTTCACGGTAACCGAA**GGTGGTGGTAGCGGTGGTGGTACTAGT**CCAAAAACAAGGAGGAGACCGCGAAGATCACAACGGAAAAGGCCGCCTACG**CCATGGCCGTAA

**scFv-sfGFP-VP64-p65-Rta-GB1-Rex NLS**

**ATGGGCCCCGACATCGTGATGACCCAGAGCCCCAGCAGCCTGAGCGCCAGCGTGGGCGACCGCGTGACCATCACCTGCCGCAGCAGCACCGGCGCCGTGACCACCAGCAACTACGCCAGCTGGGTGCAGGAGAAGCCCGGCAAGCTGTTCAAGGGCCTGATCGGCGGCACCAACAACCGCGCCCCCGGCGTGCCCAGCCGCTTCAGCGGCAGCCTGATCGGCGACAAGGCCACCCTGACCATCAGCAGCCTGCAGCCCGAGGACTTCGCCACCTACTTCTGCGCCCTGTGGTACAGCAACCACTGGGTGTTCGGCCAGGGCACCAAGGTGGAGCTGAAGCGCGGCGGCGGCGGCAGCGGCGGCGGCGGCAGCGGCGGCGGCGGCAGCAGCGGCGGCGGCAGCGAGGTGAAGCTGCTGGAGAGCGGCGGCGGCCTGGTGCAGCCCGGCGGCAGCCTGAAGCTGAGCTGCGCCGTGAGCGGCTTCAGCCTGACCGACTACGGCGTGAACTGGGTGCGCCAGGCCCCCGGCCGCGGCCTGGAGTGGATCGGCGTGATCTGGGGCGACGGCATCACCGACTACAACAGCGCCCTGAAGGACCGCTTCATCATCAGCAAGGACAACGGCAAGAACACCGTGTACCTGCAGATGAGCAAGGTGCGCAGCGACGACACCGCCCTGTACTACTGCGTGACCGGCCTGTTCGACTACTGGGGCCAGGGCACCCTGGTGACCGTGAGCAGCTACCCATACGATGTTCCAGATTACGCTGGTGGAGGCGGAGGTTCTGGGGGAGGAGGTAGTGGCGGTGGTGGTTCAGGAGGCGGCGGAAGCTTGGATCCAGGTGGAGGTGGAAGCGGTAGCAAAGGAGAAGAACTTTTCACTGGAGTTGTCCCAATTCTTGTTGAATTAGATGGTGATGTTAATGGGCACAAATTTTCTGTCCGTGGAGAGGGTGAAGGTGATGCTACAAACGGAAAACTCACCCTTAAATTTATTTGCACTACTGGAAAACTACCTGTTCCGTGGCCAACACTTGTCACTACTCTGACCTATGGTGTTCAATGCTTTTCCCGTTATCCGGATCACATGAAACGGCATGACTTTTTCAAGAGTGCCATGCCCGAAGGTTATGTACAGGAACGCACTATATCTTTCAAAGATGACGGGACCTACAAGACGCGTGCTGAAGTCAAGTTTGAAGGTGATACCCTTGTTAATCGTATCGAGTTAAAGGGTATTGATTTTAAAGAAGATGGAAACATTCTTGGACACAAACTCGAGTACAACTTTAACTCACACAATGTATACATCACGGCAGACAAACAAAAGAATGGAATCAAAGCTAACTTCAAAATTCGCCACAACGTTGAAGATGGTTCCGTTCAACTAGCAGACCATTATCAACAAAATACTCCAATTGGCGATGGCCCTGTCCTTTTACCAGACAACCATTACCTGTCGACACAATCTGTCCTTTCGAAAGATCCCAACGAAAAGCGTGACCACATGGTCCTTCTTGAGTTTGTAACTGCTGCTGGGATTACACATGGCATGGATGAGCTCTACAAA**GGTGGAGGTCGGACCGGTGGCGGTGGCGGAGGG**GACGCATTGGACGATTTTGATCTGGATATGCTGGGAAGTGACGCCCTCGATGATTTTGACCTTGACATGCTTGGTTCGGATGCCCTTGATGACTTTGACCTCGACATGCTCGGCAGTGACGCCCTTGATGATTTCGACCTGGACATGCTG**AGTTCCGGATCTCCGAAAAAGAAACGCAAAGTTGGTAGCCAGTACCTGCCCGACACCGACGACCGGCACCGGATCGAGGAAAAGCGGAAGCGGACCTACGAGACATTCAAGAGCATCATGAAGAAGTCCCCCTTCAGCGGCCCCACCGACCCTAGACCTCCACCTAGAAGAATCGCCGTGCCCAGCAGATCCAGCGCCAGCGTGCCAAAACCTGCCCCCCAGCCTTACCCCTTCACCAGCAGCCTGAGCACCATCAACTACGACGAGTTCCCTACCATGGTGTTCCCCAGCGGCCAGATCTCTCAGGCCTCTGCTCTGGCTCCAGCCCCTCCTCAGGTGCTGCCTCAGGCTCCTGCTCCTGCACCAGCTCCAGCCATGGTGTCTGCACTGGCTCAGGCACCAGCACCCGTGCCTGTGCTGGCTCCTGGACCTCCACAGGCTGTGGCTCCACCAGCCCCTAAA**CCTACACAGGCCGGCGAGGGCACACTGTCTGAAGCTCTGCTGCAGCTGCAGTTCGACGACGAGGATCTGGGAGCCCTGCTGGGAAACAGCACCGATCCTGCCGTGTTCACCGACCTGGCCAGCGTGGACAACAGCGAGTTCCAGCAGCTGCTGAACCAGGGCATCCCTGTGGCCCCTCACACCACCGAGCCCATGCTGATGGAATACCCCGAGGCCATCACCCGGCTCGTGACAGGCGCTCAGAGGCCTCCTGATCCAGCTCCTGCCCCTCTGGGAGCACCAGGCCTGCCTAATGGACTGCTGTCTGGCGACGAGGACTTCAGCTCTATCGCCGATATGGATTTCTCAGCCTTGCTG**GGCTCTGGCAGCGGCAGC**CGGGATTCCAGGGAAGGGATGTTTTTGCCGAAGCCTGAGGCCGGCTCCGCTATTAGTGACGTGTTTGAGGGCCGCGAGGTGTGCCAGCCAAAACGAATCCGGCCATTTCATCCTCCAGGAAGTCCATGGGCCAACCGCCCACTCCCCGCCAGCCTCGCACCAACACCAACCGGTCCAGTACATGAGCCAGTCGGGTCACTGACCCCGGCACCAGTCCCTCAGCCACTGGATCCAGCGCCCGCAGTGACTCCCGAGGCCAGTCACCTGTTGGAGGATCCCGATGAAGAAACGAGCCAGGCTGTCAAAGCCCTTCGGGAGATGGCCGATACTGTGATTCCCCAGAAGGAAGAGGCTGCAATCTGTGGCCAAATGGACCTTTCCCATCCGCCCCCAAGGGGCCATCTGGATGAGCTGACAACCACACTTGAGTCCATGACCGAGGATCTGAACCTGGACTCACCCCTGACCCCGGAATTGAACGAGATTCTGGATACCTTCCTGAACGACGAGTGCCTCTTGCATGCCATGCATATCAGCACAGGACTGTCCATCTTCGACACATCTCTGTTT**GGTAGTGGAGGAGGATCTCGGACCGAAGAG**TACAAGCTTATCCTGAACGGTAAAACCCTGAAAGGTGAAACCACCACCGAAGCTGTTGACGCTGCTACCGCGGAAAAAGTTTTCAAACAGTACGCTAACGACAACGGTGTTGACGGTGAATGGACCTACGACGACGCTACCAAAACCTTCACGGTAACCGAA**GGTGGTGGTAGCGGTGGTGGTACTAGT**CCAAAAACAAGGAGGAGACCGCGAAGATCACAACGGAAAAGGCCGCCTACG**CCATGGCCGTAA

**MS2-p65-HSF1**

**GCTTCAAACTTTACTCAGTTCGTGCTCGTGGACAATGGTGGGACAGGGGATGTGACAGTGGCTCCTTCTAATTTCGCTAATGGGGTGGCAGAGTGGATCAGCTCCAACTCACGGAGCCAGGCCTACAAGGTGACATGCAGCGTCAGGCAGTCTAGTGCCCAGAAGAGAAAGTATACCATCAAGGTGGAGGTCCCCAAAGTGGCTACCCAGACAGTGGGCGGAGTCGAACTGCCTGTCGCCGCTTGGAGGTCCTACCTGAACATGGAGCTCACTATCCCAATTTTCGCTACCAATTCTGACTGTGAACTCATCGTGAAGGCAATGCAGGGGCTCCTCAAAGACGGTAATCCTATCCCTTCCGCCATCGCCGCTAACTCAGGTATCTAC**AGCGCTGGAGGAGGTGGAAGCGGAGGAGGAGGAAGCGGAGGAGGAGGTAGCGGACCTAAGAAAAAGAGGAAGGTGGCGGCCGCTGGATCC**CCTTCAGGGCAGATCAGCAACCAGGCCCTGGCTCTGGCCCCTAGCTCCGCTCCAGTGCTGGCCCAGACTATGGTGCCCTCTAGTGCTATGGTGCCTCTGGCCCAGCCACCTGCTCCAGCCCCTGTGCTGACCCCAGGACCACCCCAGTCACTGAGCGCTCCAGTGCCCAAGTCTACACAGGCCGGCGAGGGGACTCTGAGTGAAGCTCTGCTGCACCTGCAGTTCGACGCTGATGAGGACCTGGGAGCTCTGCTGGGGAACAGCACCGATCCCGGAGTGTTCACAGATCTGGCCTCCGTGGACAACTCTGAGTTTCAGCAGCTGCTGAATCAGGGCGTGTCCATGTCTCATAGTACAGCCGAACCAATGCTGATGGAGTACCCCGAAGCCATTACCCGGCTGGTGACCGGCAGCCAGCGGCCCCCCGACCCCGCTCCAACTCCCCTGGGAACCAGCGGCCTGCCTAATGGGCTGTCCGGAGATGAAGACTTCTCAAGCATCGCTGATATGGACTTTAGTGCCCTGCTGTCACAGATTTCCTCT**AGTGGGCAGGGAGGAGGTGGAAGC**GGCTTCAGCGTGGACACCAGTGCCCTGCTGGACCTGTTCAGCCCCTCGGTGACCGTGCCCGACATGAGCCTGCCTGACCTTGACAGCAGCCTGGCCAGTATCCAAGAGCTCCTGTCTCCCCAGGAGCCCCCCAGGCCTCCCGAGGCAGAGAACAGCAGCCCGGATTCAGGGAAGCAGCTGGTGCACTACACAGCGCAGCCGCTGTTCCTGCTGGACCCCGGCTCCGTGGACACCGGGAGCAACGACCTGCCGGTGCTGTTTGAGCTGGGAGAGGGCTCCTACTTCTCCGAAGGGGACGGCTTCGCCGAGGACCCCACCATCTCCCTGCTGACAGGCTCGGAGCCTCCCAAAGCCAAGGACCCCACTGTCTCC**TAA

**MS2-VP64-p65-Rta**

**GCTTCAAACTTTACTCAGTTCGTGCTCGTGGACAATGGTGGGACAGGGGATGTGACAGTGGCTCCTTCTAATTTCGCTAATGGGGTGGCAGAGTGGATCAGCTCCAACTCACGGAGCCAGGCCTACAAGGTGACATGCAGCGTCAGGCAGTCTAGTGCCCAGAAGAGAAAGTATACCATCAAGGTGGAGGTCCCCAAAGTGGCTACCCAGACAGTGGGCGGAGTCGAACTGCCTGTCGCCGCTTGGAGGTCCTACCTGAACATGGAGCTCACTATCCCAATTTTCGCTACCAATTCTGACTGTGAACTCATCGTGAAGGCAATGCAGGGGCTCCTCAAAGACGGTAATCCTATCCCTTCCGCCATCGCCGCTAACTCAGGTATCTAC**AGCGCTGGAGGAGGTGGAAGCGGAGGAGGAGGAAGCGGAGGAGGAGGTAGCGGACCTAAGAAAAAGAGGAAGGTGGCGGCCGCTGGATCC**GACGCATTGGACGATTTTGATCTGGATATGCTGGGAAGTGACGCCCTCGATGATTTTGACCTTGACATGCTTGGTTCGGATGCCCTTGATGACTTTGACCTCGACATGCTCGGCAGTGACGCCCTTGATGATTTCGACCTGGACATGCTG**AGTTCCGGATCTCCGAAAAAGAAACGCAAAGTTGGTAGCCAGTACCTGCCCGACACCGACGACCGGCACCGGATCGAGGAAAAGCGGAAGCGGACCTACGAGACATTCAAGAGCATCATGAAGAAGTCCCCCTTCAGCGGCCCCACCGACCCTAGACCTCCACCTAGAAGAATCGCCGTGCCCAGCAGATCCAGCGCCAGCGTGCCAAAACCTGCCCCCCAGCCTTACCCCTTCACCAGCAGCCTGAGCACCATCAACTACGACGAGTTCCCTACCATGGTGTTCCCCAGCGGCCAGATCTCTCAGGCCTCTGCTCTGGCTCCAGCCCCTCCTCAGGTGCTGCCTCAGGCTCCTGCTCCTGCACCAGCTCCAGCCATGGTGTCTGCACTGGCTCAGGCACCAGCACCCGTGCCTGTGCTGGCTCCTGGACCTCCACAGGCTGTGGCTCCACCAGCCCCTAAA**CCTACACAGGCCGGCGAGGGCACACTGTCTGAAGCTCTGCTGCAGCTGCAGTTCGACGACGAGGATCTGGGAGCCCTGCTGGGAAACAGCACCGATCCTGCCGTGTTCACCGACCTGGCCAGCGTGGACAACAGCGAGTTCCAGCAGCTGCTGAACCAGGGCATCCCTGTGGCCCCTCACACCACCGAGCCCATGCTGATGGAATACCCCGAGGCCATCACCCGGCTCGTGACAGGCGCTCAGAGGCCTCCTGATCCAGCTCCTGCCCCTCTGGGAGCACCAGGCCTGCCTAATGGACTGCTGTCTGGCGACGAGGACTTCAGCTCTATCGCCGATATGGATTTCTCAGCCTTGCTGGGCTCTGGCAGCGGCAGCCGGGATTCCAGGGAAGGGATGTTTTTGCCGAAGCCTGAGGCCGGCTCCGCTATTAGTGACGTGTTTGAGGGCCGCGAGGTGTGCCAGCCAAAACGAATCCGGCCATTTCATCCTCCAGGAAGTCCATGGGCCAACCGCCCACTCCCCGCCAGCCTCGCACCAACACCAACCGGTCCAGTACATGAGCCAGTCGGGTCACTGACCCCGGCACCAGTCCCTCAGCCACTGGATCCAGCGCCCGCAGTGACTCCCGAGGCCAGTCACCTGTTGGAGGATCCCGATGAAGAAACGAGCCAGGCTGTCAAAGCCCTTCGGGAGATGGCCGATACTGTGATTCCCCAGAAGGAAGAGGCTGCAATCTGTGGCCAAATGGACCTTTCCCATCCGCCCCCAAGGGGCCATCTGGATGAGCTGACAACCACACTTGAGTCCATGACCGAGGATCTGAACCTGGACTCACCCCTGACCCCGGAATTGAACGAGATTCTGGATACCTTCCTGAACGACGAGTGCCTCTTGCATGCCATGCATATCAGCACAGGACTGTCCATCTTCGACACATCTCTGTTT**TAA

***CDH1* (-745-+124)-Luc2**

**TGCACTCCAGCCTGGGCAAGACAGAGCGAGACTCCGTCTCAAAAAATACAAACAAAACAAACAAACAAAAAATTAGGCTGCTAGCTCAGTGGCTCATGGCTCACACCTGAAATCCTAGCACTTTGGGAGGCCAAGGCAGGAGGATCGCTTCAGCCCAGGAGTTCGAGACCAGGCTGGGCAATACAGGGAGACACAGCGCCCCCACTGCCCCTGTCCGCCCCGACTTGTCTCTCTACAAAAAGGCAAAAGAAAAAAAAATTAGCCTGGCGTGGTGGTGTGCACCTGTACTCCCAGCTACTAGAGAGGCTGGGGCCAGAGGACCGCTTGAGCCCAGGAGTTCGAGGCTGCAGTGAGCTGTGATCGCACCACTGCACTCCAGCTTGGGTGAAAGAGTGAGACCCCATCTCCAAAACGAACAAACAAAAAATCCCAAAAAACAAAAGAACTCAGCCAAGTGTAAAAGCCCTTTCTGATCCCAGGTCTTAGTGAGCCACCGGCGGGGCTGGGATTCGAACCCAGTGGAATCAGAACCGTGCAGGTCCCATAACCCACCTAGACCCTAGCAACTCCAGGCTAGAGGGTCACCGCGTCTATGCGAGGCCGGGTGGGCGGGCCGTCAGCTCCGCCCTGGGGAGGGGTCCGCGCTGCTGATTGGCTGTGGCCGGCAGGTGAACCCTCAGCCAATCAGCGGTACGGGGGGCGGTGCCTCCGGGGCTCACCTGGCTGCAGCCACGCACCCCCTCTCAGTGGCGTCGGAACTGCAAAGCACCTGTGAGCTTGCGGAAGTCAGTTCAGACTCCAGCCCGCTCCAGCCCGGCCCGACCCGACCGCACCCGGCGCCTGCCCTCGCTCGGCGTCCCCGGCCAGCCATGGAAGATGCCAAAAACATTAAGAAGGGCCCAGCGCCATTCTACCCACTCGAAGACGGGACCGCCGGCGAGCAGCTGCACAAAGCCATGAAGCGCTACGCCCTGGTGCCCGGCACCATCGCCTTTACCGACGCACATATCGAGGTGGACATTACCTACGCCGAGTACTTCGAGATGAGCGTTCGGCTGGCAGAAGCTATGAAGCGCTATGGGCTGAATACAAACCATCGGATCGTGGTGTGCAGCGAGAATAGCTTGCAGTTCTTCATGCCCGTGTTGGGTGCCCTGTTCATCGGTGTGGCTGTGGCCCCAGCTAACGACATCTACAACGAGCGCGAGCTGCTGAACAGCATGGGCATCAGCCAGCCCACCGTCGTATTCGTGAGCAAGAAAGGGCTGCAAAAGATCCTCAACGTGCAAAAGAAGCTACCGATCATACAAAAGATCATCATCATGGATAGCAAGACCGACTACCAGGGCTTCCAAAGCATGTACACCTTCGTGACTTCCCATTTGCCACCCGGCTTCAACGAGTACGACTTCGTGCCCGAGAGCTTCGACCGGGACAAAACCATCGCCCTGATCATGAACAGTAGTGGCAGTACCGGATTGCCCAAGGGCGTAGCCCTACCGCACCGCACCGCTTGTGTCCGATTCAGTCATGCCCGCGACCCCATCTTCGGCAACCAGATCATCCCCGACACCGCTATCCTCAGCGTGGTGCCATTTCACCACGGCTTCGGCATGTTCACCACGCTGGGCTACTTGATCTGCGGCTTTCGGGTCGTGCTCATGTACCGCTTCGAGGAGGAGCTATTCTTGCGCAGCTTGCAAGACTATAAGATTCAATCTGCCCTGCTGGTGCCCACACTATTTAGCTTCTTCGCTAAGAGCACTCTCATCGACAAGTACGACCTAAGCAACTTGCACGAGATCGCCAGCGGCGGGGCGCCGCTCAGCAAGGAGGTAGGTGAGGCCGTGGCCAAACGCTTCCACCTACCAGGCATCCGCCAGGGCTACGGCCTGACAGAAACAACCAGCGCCATTCTGATCACCCCCGAAGGGGACGACAAGCCTGGCGCAGTAGGCAAGGTGGTGCCCTTCTTCGAGGCTAAGGTGGTGGACTTGGACACCGGTAAGACACTGGGTGTGAACCAGCGCGGCGAGCTGTGCGTCCGTGGCCCCATGATCATGAGCGGCTACGTTAACAACCCCGAGGCTACAAACGCTCTCATCGACAAGGACGGCTGGCTGCACAGCGGCGACATCGCCTACTGGGACGAGGACGAGCACTTCTTCATCGTGGACCGGCTGAAGAGCCTGATCAAATACAAGGGCTACCAGGTAGCCCCAGCCGAACTGGAGAGCATCCTGCTGCAACACCCCAACATCTTCGACGCCGGGGTCGCCGGCCTGCCCGACGACGATGCCGGCGAGCTGCCCGCCGCAGTCGTCGTGCTGGAACACGGTAAAACCATGACCGAGAAGGAGATCGTGGACTATGTGGCCAGCCAGGTTACAACCGCCAAGAAGCTGCGCGGTGGTGTTGTGTTCGTGGACGAGGTGCCTAAAGGACTGACCGGCAAGTTGGACGCCCGCAAGATCCGCGAGATTCTCATTAAGGCCAAGAAGGGCGGCAAGATCGCCGTGAATTCT**TAA

***RANKL* (-702-+149)-Luc2**

**TACATCTCTTTCCTGACTGTTGGGTGAGCCCTCCTCGGATGCTTGCTTCTGGCTACACGCCCCTTTACCCTTTTCTCTGCACTGTTTTCATCTTTATAAAGTCAGAGTTGGTGTCTATAGGCTCTCTACTGCCACATTCAAGACCTGCCTCGCTCAATGTCACCTTCAAGATGCAGAAATAGGGATTTGGGAAGGGGATTGTGAAATTTTCGAAGTCTTCCAAAATACTTTGAGAAACTATATTTGGAAGCACTTTGGGGGGAGAGGTTGGACAGGAAGGGTCTTCAGAGATCATCAAATTTAACTTTCTAAATCCTAAGGAGGAAACCGAGACTCCAGGATGTGAAGTCCCTTCTCTACCAAACTAGAATGGATGCAGGAGGAATGTCTGAGGTGCAATCCTTATCCTTTAGCAAAGGTGTCCTCTGCGTCTTCTTTAACCCATCTCTTGGACCTCCAGAAAGACAGCTGAGGATGGCAAGGGGAGTCTGGAACCACTGGAGTAGCCCCCAGCCTCCTCCTTGGAGGGCCCCCATGAAGGAGGCCCTTCAGTGACAGAGATTGAGAGAGAGGGAGGGCGAAAGGAAGGAAGGGGAGCCAGAGGTGGGAGTGGAAGAGGCAGCCTCGCCTGGGGCTGATTGGCTCCCGAGGCCAGGGCTCTCCAAGCGGTTTATAAGAGTTGGGGCTGCCGGGCGCCCTGCCCGCTCGCCCGCGCGCCCCAGGACCCAAAGCCGGGCTCCAAGTCGGCGCCCCACGTCGAGGCTCCGCCGCAGCCTCCGGAGTTGGCCGCAGACAAGAAGGGGAGGGAGCGGGAGAGGGAGGAGAGCTCCGAAGCGAGAGGGCCGAGCGCCATGGAAGATGCCAAAAACATTAAGAAGGGCCCAGCGCCATTCTACCCACTCGAAGACGGGACCGCCGGCGAGCAGCTGCACAAAGCCATGAAGCGCTACGCCCTGGTGCCCGGCACCATCGCCTTTACCGACGCACATATCGAGGTGGACATTACCTACGCCGAGTACTTCGAGATGAGCGTTCGGCTGGCAGAAGCTATGAAGCGCTATGGGCTGAATACAAACCATCGGATCGTGGTGTGCAGCGAGAATAGCTTGCAGTTCTTCATGCCCGTGTTGGGTGCCCTGTTCATCGGTGTGGCTGTGGCCCCAGCTAACGACATCTACAACGAGCGCGAGCTGCTGAACAGCATGGGCATCAGCCAGCCCACCGTCGTATTCGTGAGCAAGAAAGGGCTGCAAAAGATCCTCAACGTGCAAAAGAAGCTACCGATCATACAAAAGATCATCATCATGGATAGCAAGACCGACTACCAGGGCTTCCAAAGCATGTACACCTTCGTGACTTCCCATTTGCCACCCGGCTTCAACGAGTACGACTTCGTGCCCGAGAGCTTCGACCGGGACAAAACCATCGCCCTGATCATGAACAGTAGTGGCAGTACCGGATTGCCCAAGGGCGTAGCCCTACCGCACCGCACCGCTTGTGTCCGATTCAGTCATGCCCGCGACCCCATCTTCGGCAACCAGATCATCCCCGACACCGCTATCCTCAGCGTGGTGCCATTTCACCACGGCTTCGGCATGTTCACCACGCTGGGCTACTTGATCTGCGGCTTTCGGGTCGTGCTCATGTACCGCTTCGAGGAGGAGCTATTCTTGCGCAGCTTGCAAGACTATAAGATTCAATCTGCCCTGCTGGTGCCCACACTATTTAGCTTCTTCGCTAAGAGCACTCTCATCGACAAGTACGACCTAAGCAACTTGCACGAGATCGCCAGCGGCGGGGCGCCGCTCAGCAAGGAGGTAGGTGAGGCCGTGGCCAAACGCTTCCACCTACCAGGCATCCGCCAGGGCTACGGCCTGACAGAAACAACCAGCGCCATTCTGATCACCCCCGAAGGGGACGACAAGCCTGGCGCAGTAGGCAAGGTGGTGCCCTTCTTCGAGGCTAAGGTGGTGGACTTGGACACCGGTAAGACACTGGGTGTGAACCAGCGCGGCGAGCTGTGCGTCCGTGGCCCCATGATCATGAGCGGCTACGTTAACAACCCCGAGGCTACAAACGCTCTCATCGACAAGGACGGCTGGCTGCACAGCGGCGACATCGCCTACTGGGACGAGGACGAGCACTTCTTCATCGTGGACCGGCTGAAGAGCCTGATCAAATACAAGGGCTACCAGGTAGCCCCAGCCGAACTGGAGAGCATCCTGCTGCAACACCCCAACATCTTCGACGCCGGGGTCGCCGGCCTGCCCGACGACGATGCCGGCGAGCTGCCCGCCGCAGTCGTCGTGCTGGAACACGGTAAAACCATGACCGAGAAGGAGATCGTGGACTATGTGGCCAGCCAGGTTACAACCGCCAAGAAGCTGCGCGGTGGTGTTGTGTTCGTGGACGAGGTGCCTAAAGGACTGACCGGCAAGTTGGACGCCCGCAAGATCCGCGAGATTCTCATTAAGGCCAAGAAGGGCGGCAAGATCGCCGTGAATTCT**TAA

**Oligonucleotides used for the sgRNA templates**

| **Oligo name** | **Sequence (5’-3’)** |
| --- | --- |
| sgRNA_CDH1_1_s | caccGAAATTAGGCTGCTAGCTCAG |
| sgRNA_CDH1_1_as | aaacCTGAGCTAGCAGCCTAATTTC |
| sgRNA_CDH1_2_s | caccGAGAGACAAGTCGGGGCGGAC |
| sgRNA_CDH1_2_as | aaacGTCCGCCCCGACTTGTCTCTC |
| sgRNA_CDH1_3_s | caccGCCCAGGTCTTAGTGAGCCAC |
| sgRNA_CDH1_3_as | aaacGTGGCTCACTAAGACCTGGGC |
| sgRNA_CDH1_4_s | caccGGAGTTGCTAGGGTCTAGGT |
| sgRNA_CDH1_4_as | aaacACCTAGACCCTAGCAACTCC |
| sgRNA_CDH1_5_s | caccGCCACAGCCAATCAGCAGCG |
| sgRNA_CDH1_5_as | aaacCGCTGCTGATTGGCTGTGGC |
| sgRNA_RANKL_1_s | caccGCCTCCTCGGATGCTTGCTTC |
| sgRNA_RANKL_1_as | aaacGAAGCAAGCATCCGAGGAGGC |
| sgRNA_RANKL_2_s | caccGCAAGGGGAGTCTGGAACCAC |
| sgRNA_RANKL_2_as | aaacGTGGTTCCAGACTCCCCTTGC |

**Primers used for the construction of MS2-*n*x22sTag expression vectors**

| **Oligo name** | **Sequence (5’-3’)** |
| --- | --- |
| 22sTag_vec_4n-V_F | ACCGCGGTAAACATAGGTGGTGG |
| 22sTag_vec_1-V_R | TTCTTCCGCGGCGTCAGTC |
| 22sTag_ins_V-1_F | gacgccgcggaaGAAGAGCTCCTTAGTAAGAACTATCATCTGG |
| 22sTag_ins_4-5_F | ggctcagggtctgggGAAGAGCTCCTTAGTAAGAACTATCATCTGG |
| 22sTag_ins_V-4n_R* | tatgtttaccgcggtGCCTGAGCCTGAGCCCTTC |
| 22sTag_ins_5-4_R | CCCAGACCCTGAGCCACC |
| 22sTag_Vec_8n-V_F | GATCTACAGCGGCCGCAAGG |
| 22sTag_Vec_16-8_R | CATCGGACCGGTTCCACCAC |
| 22sTag_Ins_8-16_F | ggaaccggtccgatggatctacagcggccgAGCAGCGGATCCAACGGTCC |
| 22sTag_Ins_24-16_R | gttggatccgctgctCGGCCGCTGTAGATCCATCG |
| 22sTag_Ins_16-24_F | AGCAGCGGATCCAACGGTCC |
| 22sTag_Ins_V-8n_R | CGGCCGCTGTAGATCCATCG |

*The design of this primer was inappropriate, but no frame-shift mutation was observed in the constructed plasmid.

**Primers used for qRT-PCR**

| **Oligo name** | **Sequence (5’-3’)** |
| --- | --- |
| CDH1_Forward | GACTCGTAACGACGTTGCAC |
| CDH1_Reverse | CCGCTTCCTTCATAGTCAAA |
| RANKL_Forward | ATCAGAGCAGAGAAAGCGATG |
| RANKL_Reverse | ACTCACTTTATGGGAACCAGATG |
| RPL8_Forward | AGGGCATCGTCAAGGACATC |
| RPL8_Reverse | CGTCCGCTTCTTAAACCGATAC |

**Synthesized 4x22sTag sequence**

GAAGAGCTCCTTAGTAAGAACTATCATCTGGAAAATGAGGTAGCGCGCTTAAAGAAAGGGTCGGGAAGTGGCGGCAGCGGAAGTGGGAGTGGAGGGAGCGGTTCTGGCGGTTCCGGCAGTGGAGAGGAGTTGCTGTCTAAGAACTACCACTTAGAAAACGAAGTCGCACGGCTAAAAAAAGGTTCTGGCTCCGGAGGCAGTGGTTCTGGAAGCGGTGGCAGCGGGTCAGGTGGAAGCGGATCAGGTGAGGAATTGCTTTCCAAAAACTACCACCTTGAGAATGAGGTGGCCAGGTTAAAGAAGGGCAGCGGCTCGGGGGGTAGTGGATCGGGGAGTGGCGGGTCAGGAAGCGGTGGTAGCGGAAGCGGGGAGGAGCTGCTCTCGAAGAATTACCATTTGGAGAACGAAGTGGCGAGACTAAAGAAGGGAAGCGGCAGTGGTGGATCTGGGTCTGGTTCAGGTGGGAGTGGGAGCGGTGGCTCAGGGTCTGGG
