# Supplementary material for: Three-Component Repurposed Technology for Enhanced Expression: Highly Accumulable Transcriptional Activators via Branched Tag Arrays
Source: CRISPR J. 2018 Oct 23;1(5):337–47. doi: 10.1089/crispr.2018.0009 (PMC6636879; doi:10.1089/crispr.2018.0009)
Supplement: Supplemental data [file Supp_Fig2.pdf]

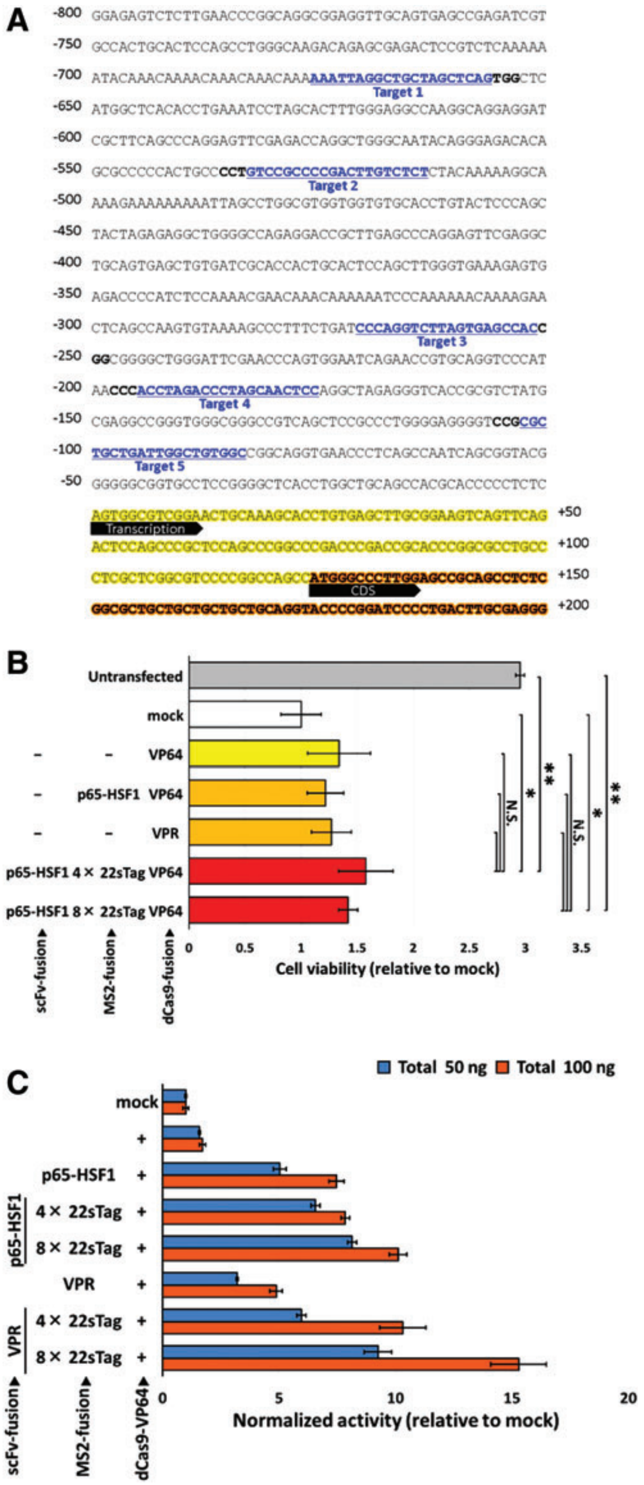

SUPPLEMENTARY FIG. S2. Supplemental data of *CDH1* activation and cytotoxicity analysis in MIA-PaCa2 cells. (**A**) Detailed design of sgRNAs used for the activation of *CDH1*, related to Figures 2A and B. (**B**) Viability of the cells transfected with the vectors shown at the left. Data are shown as the mean ± S.D. ($n = 4$). (**C**) Dose response of the vectors transfected (50 ng vs. 100 ng in total), related to Figure 2E. Data are shown as the mean ± S.D. ($n = 4$). N.S., not significant.
