# Supplementary material for: Three-Component Repurposed Technology for Enhanced Expression: Highly Accumulable Transcriptional Activators via Branched Tag Arrays
Source: CRISPR J. 2018 Oct 23;1(5):337–47. doi: 10.1089/crispr.2018.0009 (PMC6636879; doi:10.1089/crispr.2018.0009)
Supplement: Supplemental data [file Supp_Fig4.pdf]

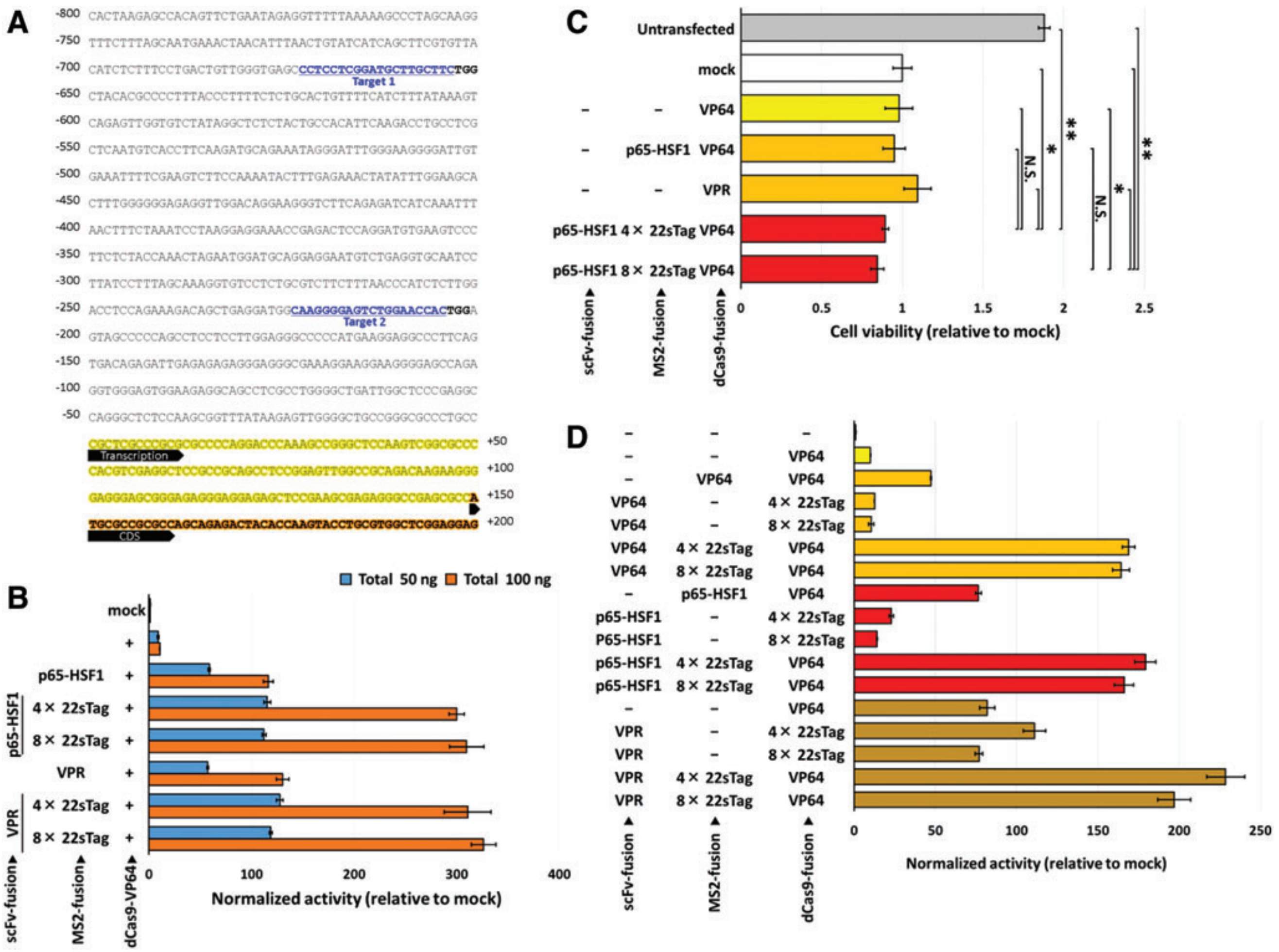

SUPPLEMENTARY FIG. S4. Supplemental data of *RANKL* activation and cytotoxicity analysis in HEK293T cells. (**A**) Detailed design of sgRNAs used for the activation of *RANKL*, related to Figures 3A and B. (**B**) Dose response of the vectors transfected (50 ng vs. 100 ng in total), related to Figure 3C. Data are shown as the mean ± S.D. ($n = 4$). (**C**) Viability of the cells transfected with the vectors shown at the left. Data are shown as the mean ± S.D. ($n = 4$). **$p < 0.01$, *$p < 0.05$. (**D**) Comparison of activity between the MS2-22sTag (TREE) and dCas9-22sTag systems, by reporter assay. For dCas9-4× and 8× 22sTag, sgRNAs without MS2 stem loops were used instead of sgRNA2.0. Data are shown as the mean ± S.D. ($n = 4$). N.S., not significant.
