# Supplementary material for: Three-Component Repurposed Technology for Enhanced Expression: Highly Accumulable Transcriptional Activators via Branched Tag Arrays
Source: CRISPR J. 2018 Oct 23;1(5):337–47. doi: 10.1089/crispr.2018.0009 (PMC6636879; doi:10.1089/crispr.2018.0009)
Supplement: Supplemental data [file Supp_Fig1.pdf]

Supplementary Data

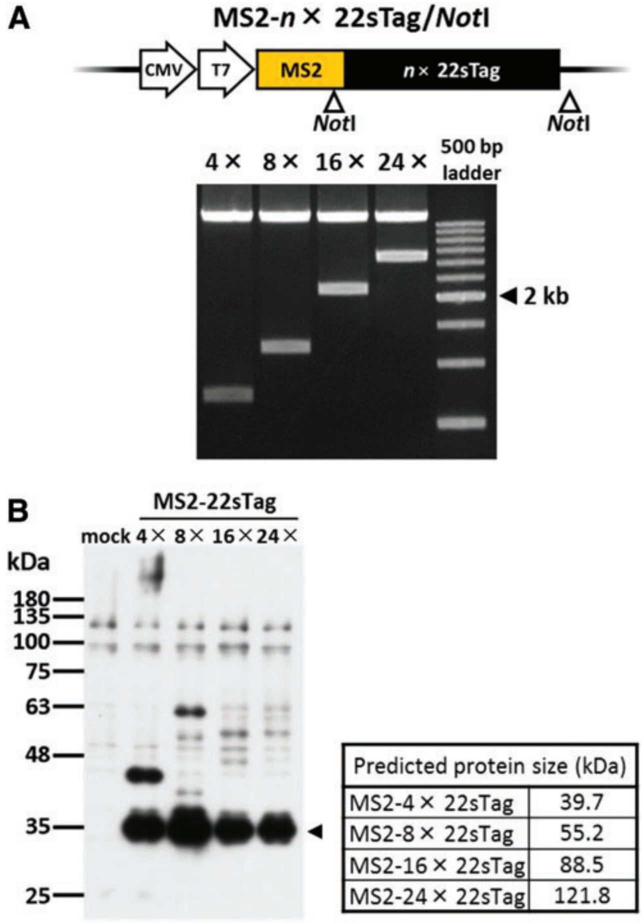

Predicted protein size (kDa)	
MS2-4 × 22sTag	39.7
MS2-8 × 22sTag	55.2
MS2-16 × 22sTag	88.5
MS2-24 × 22sTag	121.8

SUPPLEMENTARY FIG. S1. Validation of MS2-22sTag constructs. (**A**) Validation of MS2-22sTag vectors by restriction digestion. A gel image of MS2-*n* × 22sTag vector digested with *Not*I is shown. The band pattern suggested successful construction of the vectors. (**B**) Western blotting analysis of MS2-*n* × 22sTag proteins expressed in HEK293T cells. 10 μg each of cell lysate was loaded and the corresponding protein was detected with anti-HA antibody. Predicted protein sizes are shown at the right table. A filled triangle indicates the bands of truncated proteins, possibly containing MS2 coat protein and partial GCN4 tag arrays.
