# Supplementary material for: Three-Component Repurposed Technology for Enhanced Expression: Highly Accumulable Transcriptional Activators via Branched Tag Arrays
Source: CRISPR J. 2018 Oct 23;1(5):337–47. doi: 10.1089/crispr.2018.0009 (PMC6636879; doi:10.1089/crispr.2018.0009)
Supplement: Supplemental data [file Supp_Fig3.pdf]

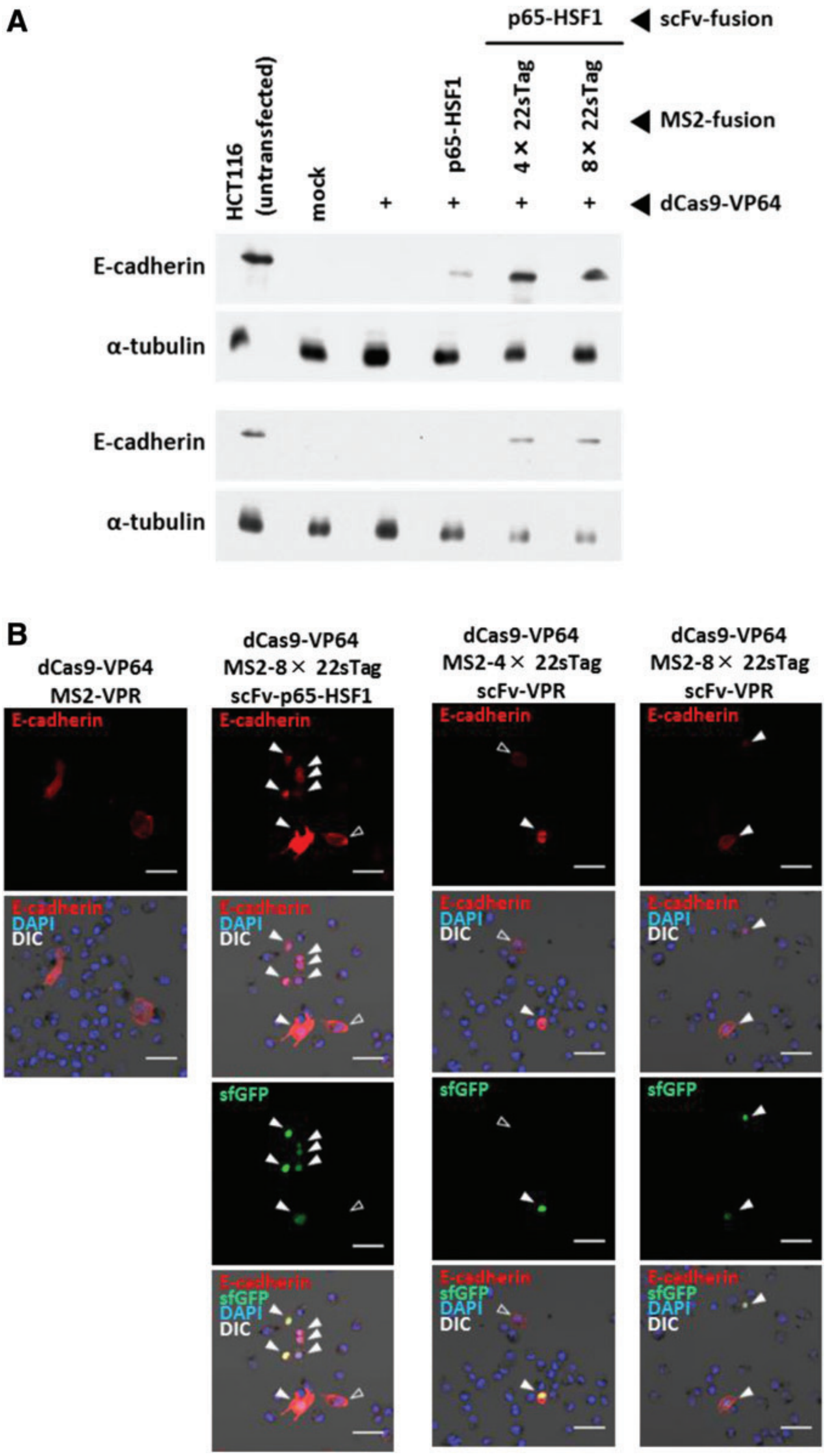

SUPPLEMENTARY FIG. S3. Supplemental data of E-cadherin protein expression. (**A**) Blots for the detection of E-cadherin and α-tubulin proteins not shown in Figure 2G. Loaded protein mass is the same as Figure 2G. (**B**) Additional fluorescence images obtained in the immunostaining experiments not shown in Figure 2H. Filled and open triangles indicate E-cadherin/sfGFP- and E-cadherin-positive cells. Scale bars, 50 μm.
